# New Light on Historical Specimens Reveals a New Species of Ladybird (Coleoptera: Coccinellidae): Morphological, Museomic, and Phylogenetic Analyses

**DOI:** 10.3390/insects11110766

**Published:** 2020-11-06

**Authors:** Karen Salazar, Romain Nattier

**Affiliations:** 1 Institut de Systématique, Evolution, Biodiversité (ISYEB), Muséum national d’Histoire naturelle, CNRS, Sorbonne Université, EPHE, Université des Antilles, 57 rue Cuvier, CP 50, 75005 Paris, France; nattier@mnhn.fr; 2 Grupo de Investigación Insectos de Colombia, Instituto de Ciencias Naturales, Universidad Nacional de Colombia, Ciudad Universitaria, Bogotá 111321, Colombia

**Keywords:** biological collections, Coleoptera, Illumina, mitogenome, molecular phylogeny, NGS, South America, taxonomy, type specimens

## Abstract

**Simple Summary:**

Biological collections are a valuable source of genetic information. Museomics in combination with morphological analysis is useful for systematic studies. *Eriopis* is a genus of ladybird beetles (Coccinellidae) that lives in South America. This study presents *Eriopis patagonia*, a new species of ladybird beetle discovered with two old specimens collected in Patagonia at least 100 years ago and deposited in a natural history collection. DNA was extracted from the specimens by a non-destructive method, allowing the specimens to be preserved again. The total gDNA was sequenced using Next-Generation Sequencing (NGS) technologies. The genetic information obtained allows us to reconstruct and describe its mitochondrial genome and examine its phylogenetic position.

**Abstract:**

Natural history collections house an important source of genetic data from yet unexplored biological diversity. Molecular data from museum specimens remain underexploited, which is mainly due to the degradation of DNA from specimens over time. However, Next-Generation Sequencing (NGS) technology can now be used to sequence “old” specimens. Indeed, many of these specimens are unique samples of nomenclatural types and can be crucial for resolving systematic or biogeographic scientific questions. Two ladybird beetle specimens from Patagonia corresponding to a new species of the genus *Eriopis* Mulsant were found in the collections of the Muséum national d’Histoire naturelle (MNHN), Paris. Here, we describe ***Eriopis patagonia*** Salazar, **sp. nov.** Total DNA of one of the two specimens was sequenced by NGS using a paired-end Illumina approach. We reconstruct and characterize the mitochondrial genome of this species (16,194 bp). Then, the protein-coding genes (PCGs) and ribosomal RNAs (rRNAs) were used to infer by maximum likelihood and Bayesian Inference the phylogenetic position of *E. patagonia* among 27 representatives of Coccinellidae. Phylogenetic analysis confirmed the position of *Eriopis* as sister group to *Cycloneda* Crotch. Hence, we highlight the high potential of sequencing technology for extracting molecular information from old specimens, which are used here for the systematic study of a genus, while demonstrating the importance of preserving biological collections.

## 1. Introduction

Natural history collections (NHCs) are spatio-temporal testimonies of biological diversity and serve as an invaluable tool for documenting changes in biodiversity over time [1,2,3]. The acquisition of genetic data from NHC specimens is made difficult by processes such as DNA degradation, DNA fragmentation, and contamination. The degradation of a specimen’s DNA over time begins postmortem and increases over time depending on storage conditions, resulting in DNA fragmentation and reducing the quality and quantity of usable DNA from older NHC specimens (reviewed in [4,5]). Next-Generation Sequencing (NGS) is a high-throughput data collection method that uses short read sequences as templates, thus making it possible to bypass the above-mentioned difficulties by sequencing the highly fragmented DNA of old specimens [6,7,8]. The use of NGS for low-coverage whole genome sequencing is called “Genome Skimming” [9,10]. This method has been successfully applied to obtain plastomes, mitogenomes, and repetitive nuclear loci from various plant (e.g., [11,12,13]) and animal groups (e.g., insects [14,15]; annelids [16]; crayfish [17]). Furthermore, non-destructive protocols allow the extraction of DNA from rare or unique samples and type specimens from NHCs. For arthropods, the most common protocol consists in placing the entire specimen in a lysis buffer to extract its DNA. This process causes no discernible external morphological damage and sample specimens can be stored again in NHCs [18,19,20,21].

Specimens kept in NHCs are often used in a traditional taxonomic context [5]. However, these specimens also contain genetic information waiting to be explored. For example, museomics has been used to resolve phylogenies (e.g., [22,23,24]), for accurate species designation using DNA from type specimens [25,26], to reveal cryptic species [27], to assess the phylogenetic relationship of extinct lineages (e.g., [28,29]), and to investigate genetic changes in populations over time [4]. Furthermore, molecular analysis of old museum specimens can also accelerate the process of species discovery [30].

Very few studies have recovered genetic information from old beetle (Coleoptera) specimens deposited at NHCs. Maddison and Cooper [31] successfully sequenced eight genes from a dry pinned specimen of the carabid *Bembidion orion* Cooper collected in 1968 and used that specimen as a reference in a species delimitation approach. Heintzman et al. [32] amplified mitochondrial and nuclear DNA fragments from several carabid *Amara alpina* (Paykull) specimens collected between 1875 and 1999. Kanda et al. [33] successfully recovered protein-coding, ribosomal, and mitochondrial genes from one 84-year-old (age before DNA extraction) tenebrionid beetle, and from four to 69-year-old specimens of the carabid beetles *Bembidion* Latreille and *Lionepha* Casey to test their phylogenetic position and identify the factors that impact the success of sequencing NHCs specimens. Sproul and Maddison [34] used dry-stored carabid specimens of *Lionepha* and *Bembidion* (ca. 159–58 years old) to amplify their mitochondrial genome, nuclear rDNA complex, and 67 low-copy-number nuclear protein-coding genes. Finally, Jin et al. [27] recovered mitochondrial genes from specimens of prionine longhorn beetles that were up to 128 years old, which allowed them to identify new taxa and provide new insights into the phylogeny of this group. All these studies explored DNA preservation in museum beetle specimens and highlighted the importance of NHCs specimens for genetic studies.

*Eriopis* Mulsant is a South American genus of ladybird beetles (Coccinellidae, Coccinellinae, Coccinellini) currently comprising 23 species [35]. Taxonomic identification at the species level of certain *Eriopis* is difficult due to similarities in body coloration design and genitalia morphology [36]. For several species and subspecies, only the type series, which may be an old specimen, is known; for others, there are doubts regarding their taxonomic position. Therefore, NGS is an attractive tool to investigate the systematics of this genus, and this study is a first step in this process. While revising *Eriopis* specimens from several American and European NHCs, we found two historical specimens collected before 1930, which correspond to a new species. The discovery of these two unique and well-preserved samples opened the possibility of DNA sequencing using the Illumina NGS platform. In this study, we (i) describe this new species and compare its morphology with related taxa, (ii) reconstruct and characterize its mitochondrial genome, and (iii) examine its phylogenetic position.

## 2. Material and Methods

### 2.1. Specimens and Taxonomy

We studied two specimens, designated here as type series, which were preserved dry and glued to a card mount and deposited in the Muséum national d’Histoire naturelle (MNHN), Paris, France. To dissect the male genitalia, the holotype was softened in a wet chamber containing distilled water, after which the abdomen was removed and the tergites and sternites were separated laterally. Dissected genitalia were cleared in a 5% cold KOH (Potassium hydroxide) solution and preserved in glass vials containing glycerin. Digital photographs of the genitalia were taken using a Canon EOS 60D Digital SLR camera on a Nikon SMZ1500 stereomicroscope at the MNHN. The genitalia terminology used in this study follows Ślipiński [37]. Holotype dissection was carried out after DNA extraction to avoid losing DNA-containing tissue and altering the quality of the genetic material with the dissection process. After DNA extraction, the re-mounted specimens were photographed with a Canon EOS 6D Digital SLR camera at the MNHN. All measurements of the beetle’s body were obtained with the image-processing package FIJI (open source) [38].

The nomenclatural acts resulting from this study follow the International Code of Zoological Nomenclature (ICZN) [39]. This published work and the nomenclatural acts have been registered in the online registration system of ZooBank (http://zoobank.org), following the Life Science Identifiers (LSIDs).

### 2.2. DNA Extraction

First, we cleaned the two specimens and removed them from the mounts following the recommendations of Kanda et al. [33]. Next, we used the non-invasive DNA extraction protocol suggested by Gilbert et al. [18], in which whole specimens (without removing body parts) are used. We observed that after the process of DNA extraction, the only visible external alterations in these *Eriopis* specimens were a lightening of the yellow spots and the loss of a few tarsi. Total genomic DNA (gDNA) was extracted in October 2018 using a QIAamp DNA Micro Kit (Qiagen Inc.) following the manufacturer’s instructions. The quantification of extracted gDNA was performed with a Qubit™ dsDNA High-Sensitivity (HS) Assay Kit with a Fluorescence Microplate Reader in a 1.0 µL sample. Only gDNA extracted from the holotype (sequence code: K22: PA-PA-H-1) was processed for sequencing. gDNA from the paratype (K47: PA-PA-P-2) was stored at −30 °C at the MNHN.

### 2.3. Library Preparation and DNA Sequencing

DNA quality and quantity metrics from the previous step were used for library preparation. Genomic DNA was indexed and libraries were prepared using the NEBNext^®^ Ultra^TM^ II DNA Library Prep Kit for Illumina (New England BioLabs), following the manufacturer’s instructions. The preparation of the Adapter Mix and Adapter Fill-In steps were performed as described by Meyer and Kircher [40]. After adapter ligation, the reaction was purified using solid phase reversible immobilization (SPRI) with Carboxyl-coated magnetic beads. Here, we selected fragments ranging from 400 to 500 base pairs (bp). Following the size selection step, real-time PCR (qPCR) (CFX 96^©^ BIORAD) was conducted to determine the optimal number of amplification cycles for PCR indexing (PCR cycling conditions were initial denaturation at 98 °C for 2 min followed by 15 cycles of denaturation at 95 °C for 30 s, primer annealing at 58 °C for 30 s, and extension at 72 °C for 40 s, with a final elongation of 5 min at 72 °C). After PCR indexing, another purification reaction was performed as above. Total gDNA was quantified with a Qubit™ dsDNA (HS) Assay Kit using Qubit^TM^ Fluorometer (Life Technologies) in a 1.0 µL sample after library preparation. Libraries were quantified with a DNA 1000 series II chip on a 2100 Bioanalyzer (Agilent Technologies, Santa Clara, CA, USA) (High Sensitivity DNA Assay). Pooled libraries were sequenced as 150 paired-end reads on an Illumina HiSeq 3000 HWI-J0015 on a single lane at the Genome and Transcriptome Platform Genotoul (Toulouse, Haute-Garonne, France).

### 2.4. Genome Assembly, Annotation, and Reconstruction

Sequencing reads from both paired-end libraries were imported in Geneious Prime 2019.1.3 (Biomatters Ltd.); then, low-quality reads and adaptor contamination were trimmed using the BBDuk plugin (minimum quality score of 30 and minimum read length of 30 bp). Quality and length distribution were inspected using FastQC v. 0.11.8 [41]. Using the “Map to Reference” option in Geneious (custom sensibility, fine tuning: iterate up to 10 times; max. mismatches per read 30), we extracted mitochondrial sequence fragments from the total reads. The mitochondrial genome of *Coccinella septempunctata* (Linnaeus) (Coccinellidae, Coccinellinae) (GenBank accession number: JQ321839) was used as reference [42]. After removing the reference sequence, de novo assembly (sensibility: high sensibility/medium) was performed in Geneious. The longest resulting contigs were chosen as seeds and were used to map the filtered reads (custom sensibility, fine-tuning: iterate up to 25 times; max. mismatches per read 10). The resulting sequence contigs were used to generate a consensus sequence and create a circular molecule. Cytochrome c oxidase subunit 1 (COX1), Cytochrome b (Cyt B), and small subunit and large subunit mitochondrial ribosomal RNAs (12S and 16S) were submitted to standard nucleotide BLAST (https://blast.ncbi.nlm.nih.gov/Blast.cgi) to corroborate the taxonomic identity of this specimen within Coleoptera Coccinellidae. The identity and position of protein-coding genes (PCGs), transfer RNA (tRNA), and rRNA genes were determined using the MITOS web server (http://mitos.bioinf.uni-leipzig.de) [43]; and tRNAscan-SE 1.21 [44], in combination with visual comparison with the annotated mitogenome of a closely related taxon (*C. septempunctata*) in Geneious, followed by manual verification. The tRNAs are labeled according to the IUPAC-IUB (International Union of Pure and Applied Chemistry-International Union of Biochemistry) amino acid code. All PCGs were translated (transl_table 5) to confirm the presence of start and stop codons and check for the absence of pseudogenes with the “translate” option in Geneious. Reconstruction of the mitochondrial genome was also performed with Geneious. Nucleotide composition was estimated as percentage and AT- and CG-skews, as (A − T)/(A + T) and (G − C)/(G + C), respectively, where G, A, T, and C are the frequencies of each nucleotide [45]. The mitochondrial genome was submitted to GenBank with the GenBank submission tool in Geneious.

### 2.5. Phylogenetic Analysis

The phylogenetic position of *E. patagonia* was inferred from 27 Coccinellidae mitochondrial genome sequences available in GenBank (https://www.ncbi.nlm.nih.gov/genbank; accessed in 20 January 2020), with *Dastarcus helophoroides* (Fairmaire) and *Gloeosoma* sp. used as outgroups (Table 1). The 13 PCGs and the two rRNA genes described above were extracted from each mitogenome and aligned separately with the MAFFT (Multiple Alignment using Fast Fourier Transform) algorithm [46,47] implemented on the online MAFFT server: http://mafft.cbrc.jp/alignment/server/large.html, under the default parameters on the server. The ambiguously aligned regions of 16S and 12S rRNAs were removed with trimAL v.1.4 [48] using automatic configuration to the heuristic approach. All resulting alignments were checked in AliView [49]. The gene dataset was concatenated with SequenceMatrix [50]. The initial molecular dataset was partitioned a priori in blocks and treated with three partitioning schemes. (1) PCG_RNA (matrix totaling 13,167 nucleotides in length; 41 blocks): one block for each rRNA, and three for each PCG corresponding to each nucleotide position in the codon (including all codon positions); (2) PCG12_RNA (9429 nucleotides in total length; 28 blocks): one for each rRNA and two for each PCG (excluding the third-codon position); (3) PCG_AA (7720 nucleotides in total length; 13 blocks): 13 PCGs were translate into amino acids. The best partitioning scheme and substitution models to each partition scheme were determined with PartitionFinder v2.1.1 [51] according to the Bayesian information criterion (BIC), with linked branch lengths and a *greedy* search algorithm [52] (Table S1A–C).

The concatenated matrices were analyzed using maximum likelihood (ML) with RAxML (Randomized Axelerated Maximum Likelihood) v8.2.12 [53] and Bayesian Inference (BI) as implemented in MrBayes 3.2.7a [54], which were both performed in the public resource CIPRES Science Gateway V. 3.3 (http://www.phylo.org) [55]. RAxML was based on the BIC (with the *RaxMl* set of models), and the GTR (general time reversible) + I (proportion of invariable sites) + G (gamma distribution) substitution model was selected for the subsets proposed by PartitionFinder. The best tree was obtained using a heuristic search implementing 100 random-addition replicates. In addition to the Felsenstein’s bootstrap (FB), we implemented the transfer bootstrap expectation (TBE) statistic with 1000 replicates and a 70% threshold in the BOOSTER web interface (available at https://booster.pasteur.fr). TBE provides a better measurement of branch repeatability or robustness [56]. The BI analysis was based on the BIC (with the *MrBayes* set of models) and was performed in two independent runs with four MCMC (Markov chain Monte Carlo) chains run for 50 million generations each, sampling values every 1000 generations. A conservative burn-in of 25% was applied after checking for convergence in Tracer v1.7.1 [57]. We assessed the convergence of the runs by investigating the average standard deviation of split frequencies and Effective Sample Size (ESS) of all parameters. The support of nodes was provided by clade posterior probabilities (PP) as directly estimated from the majority-rule consensus topology.

Graphical representation of the trees were made using iTOL [58] and edited in Omnigraffle 7.2.10 (https://www.omnigroup.com/omnigraffle/).

### 2.6. Estimation of Evolutionary Divergence between Sequences

We compared the level of mitogenome divergence (PCGs and PCGs + rRNAs) between the new *Eriopis* species and the other Coccinelloidea included in this study (Table 1). Analyses were carried out using the Kimura 2-parameter model. The variation rate among sites was modeled with a gamma distribution (shape parameter = 1). The 1st, 2nd, and 3rd codon positions were included. All positions with less than 95% site coverage were eliminated; i.e., less than 5% alignment gaps, missing data, and ambiguous bases were allowed at any position (partial deletion option). Evolutionary analyses were conducted in MEGA X [59,60].

## 3. Results

### Taxonomy

*Eriopis* Mulsant, 1850.

***Eriopispatagonia*** Salazar, **sp. nov.**

**Figure 1:**
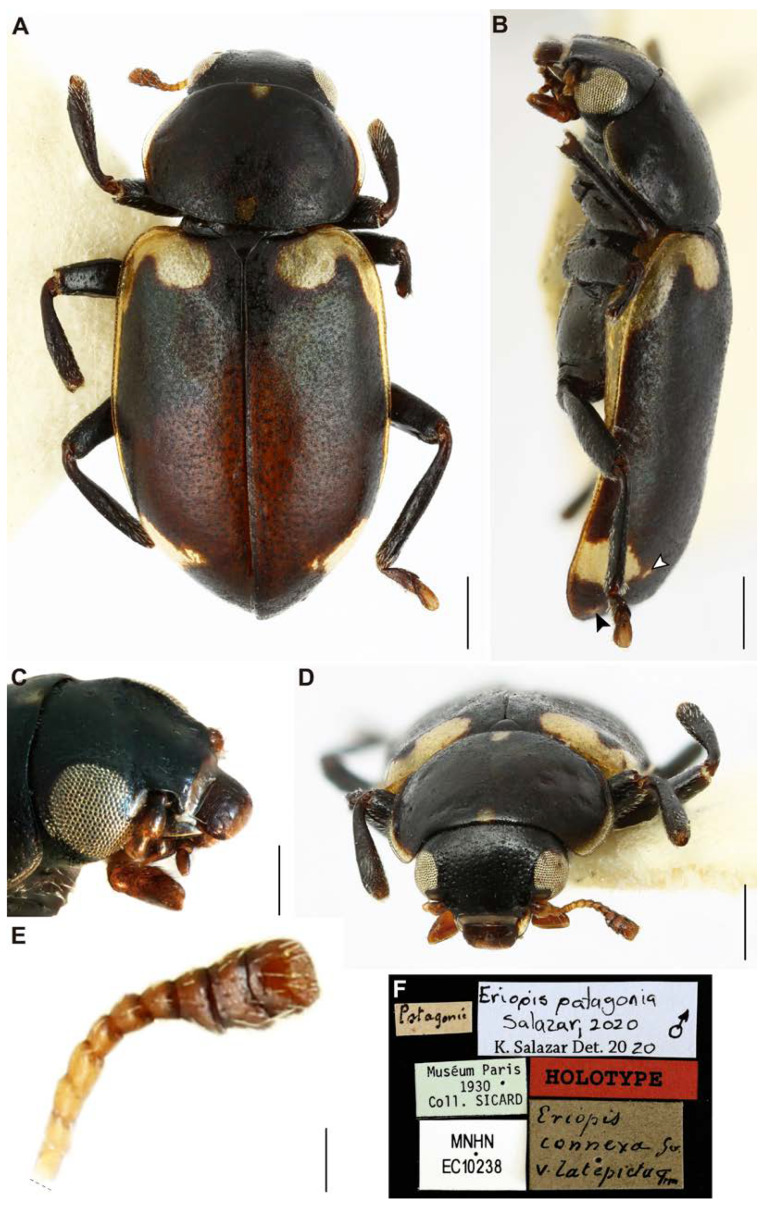
*Eriopis patagonia* Salazar, sp. nov. — Holotype: dorsal (**A**), lateral (**B**,**C**) and frontal (**D**) views. (**B**). The arrowheads signaled the tiny additional spots, which are not present in the Paratype. (**E**). Antenna with 10 antennomeres (without the basal scape). (**F**). Labels. Photographs of the specimen were made after DNA extraction. Scale bars (mm): (**A**–**D**) 0.5; (**E**) 0.12.

**Figure 2:**
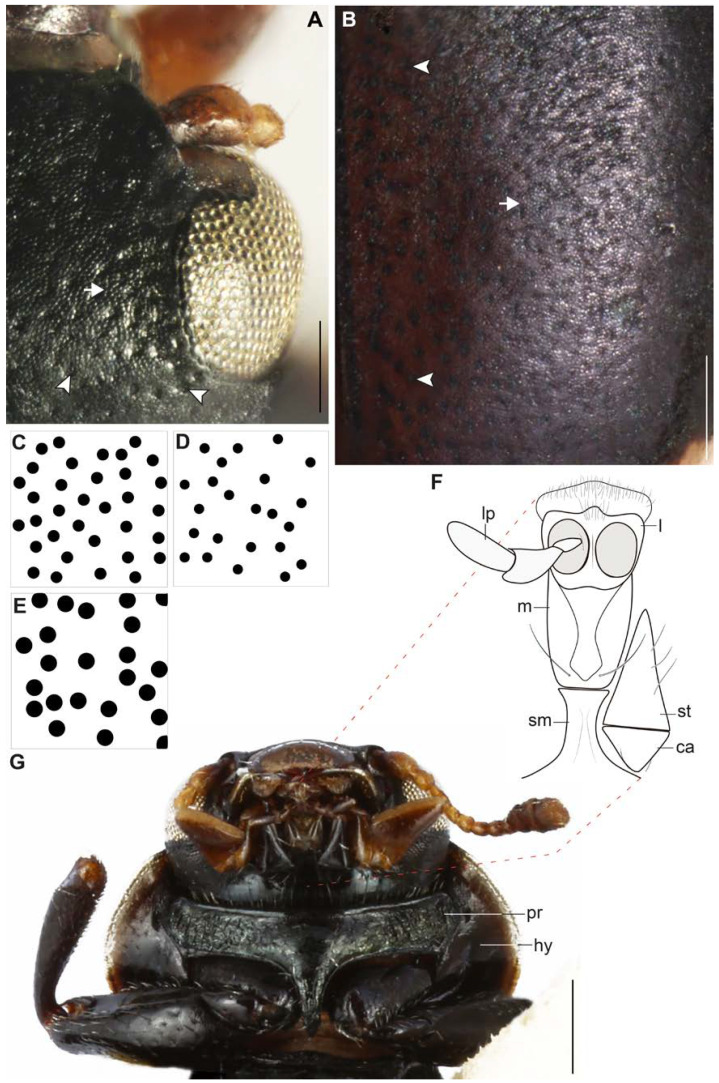
*Eriopis patagonia* Salazar, sp. nov. **—** Holotype. (**A**,**C**,**F**,**G**). Head. (**D**)**.** Pronotum. (**B**,**E**). Elytron. Observe in (**A**) and (**B**) the punctuations (arrowheads) and micropunctuations (arrows). (**C**–**E**). Dorsal punctuation of the body in squares of 0.25 × 0.25 mm. Note the difference in the size. (**F**). Submenton (sm), menton (m), ligule (l), labial palp (lp), cardo (ca), and stipe (st). (**G**). Ventral view of head and thorax. Dotted lines signal the representation in (**F**). pr: prosternum; hy: hypomeron. Scale bars (mm): (**A**,**B**) 0.12; (**G**) 0.25.

**Figure 3:**
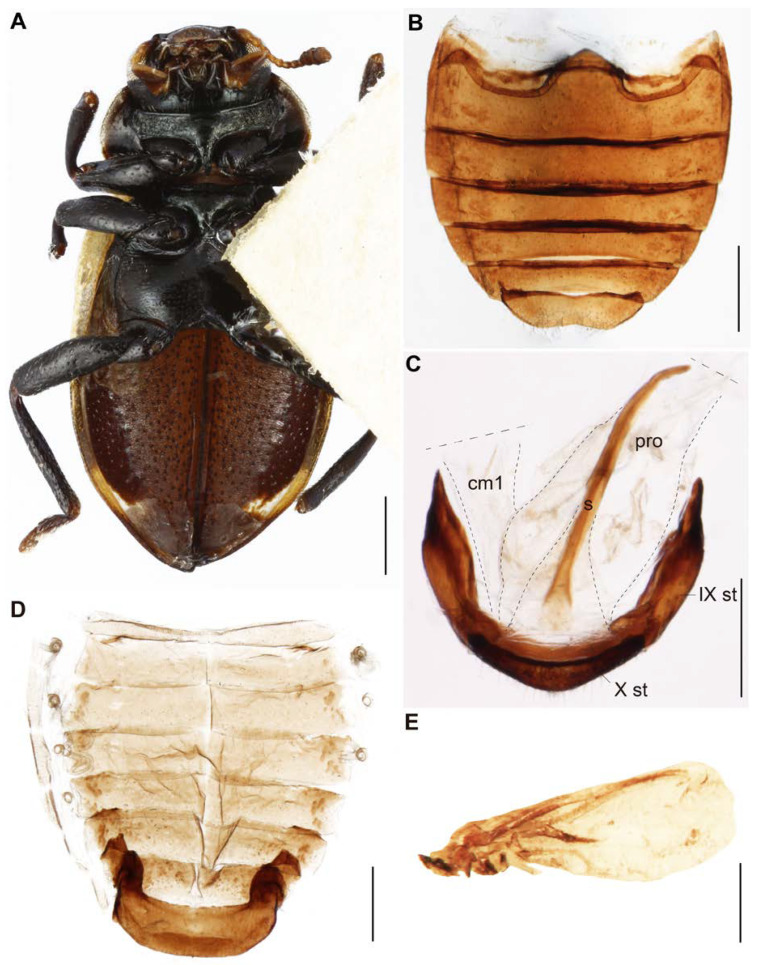
*Eriopis patagonia* Salazar, sp. nov. —Holotype. (**A**). In ventral view. (**B**,**C**). Sternites (st) III–VIII (**B**) and IX–X (**C**). (**D**). Tergites. cm1: connective membrane 1; pro: proctodeum; s: spicule. (**E**). Left hindwing. Dotted lines in (**C**) indicate membrane structures. Scale bars (mm): (**A**,**B**,**D**) 0.5; (**C**,**E**) 0.25.

**Figure 4:**
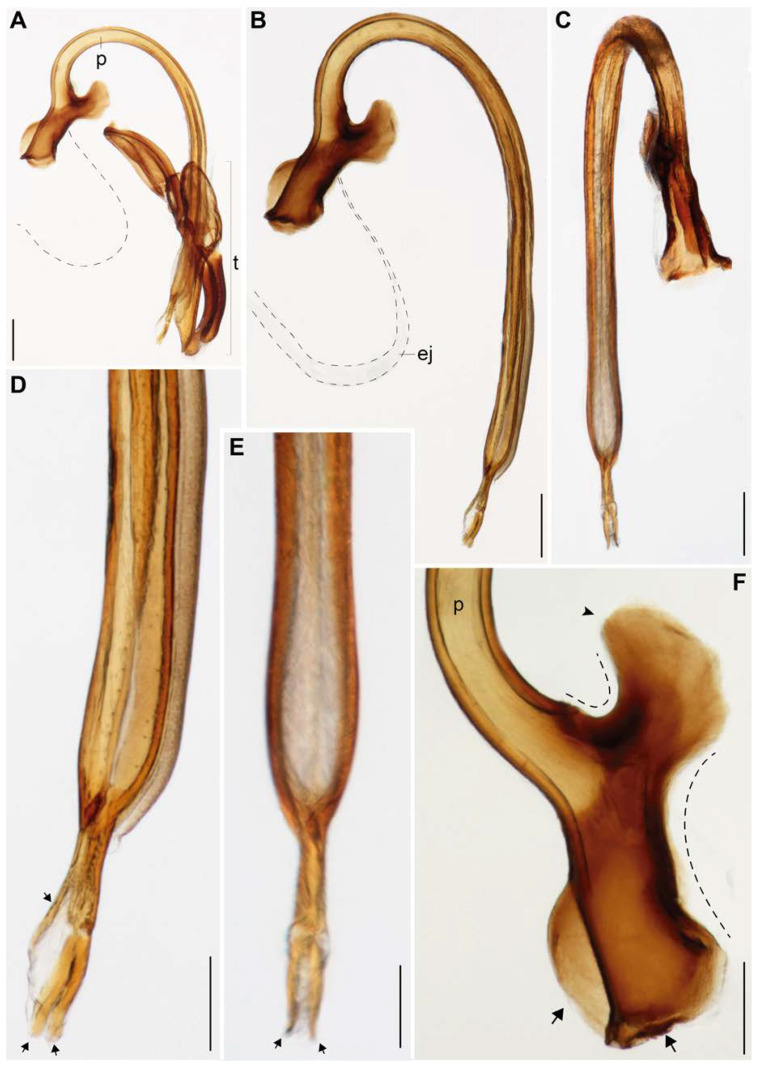
Male genitalia of *Eriopis patagonia* Salazar, sp. nov**. —** Holotype: (**A**,**B**,**D**,**F**) lateral and (**C**,**E**) ventral views. (**A**). Complete genitalia. Compare the relation between the size of the penis (p) and the tegmen (t). (**B**–**F**). Penis separated from the t. (**D**,**E**). The distal region of the p. Observe the proportion between the three apical structures (arrows). (**F**). The most proximal region of the p (penis capsule). Observe the two lateral extensions: the inner one (arrowhead) and outer one, which is divided into two parts (arrows). ej: ejaculator duct. Dotted lines in (**A**,**B**) indicate membrane structures and in (**F**) concavity formed between the structures. Scale bars (mm): (**A**–**C**) 0.25; (**D**–**F**) 0.12.

**Figure 5:**
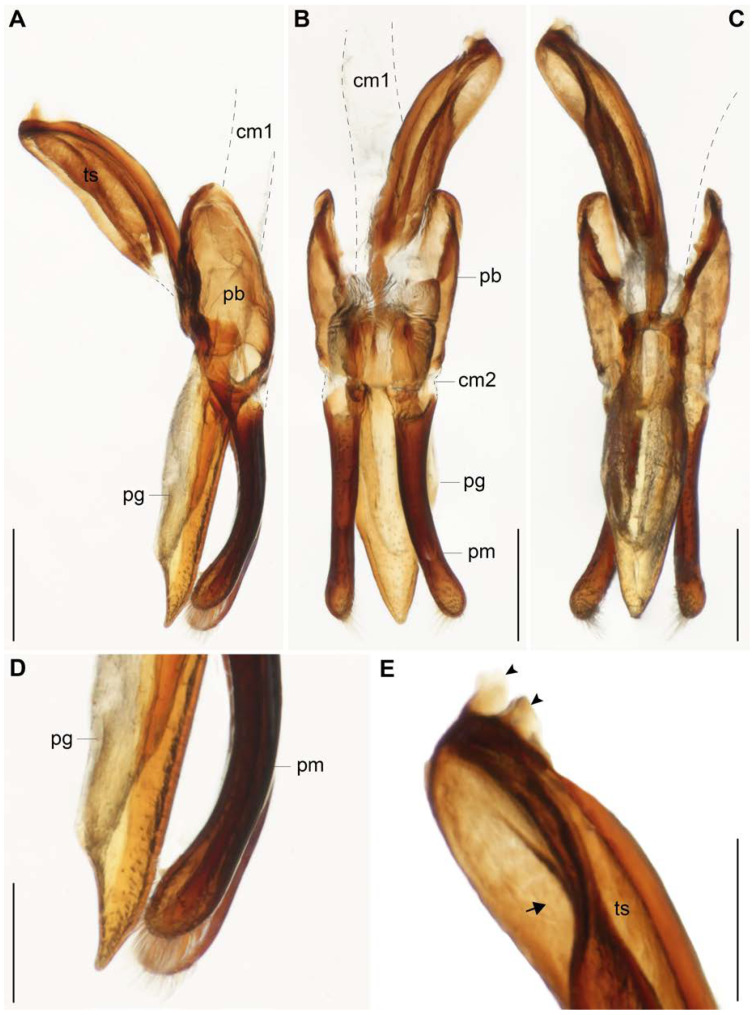
Male genitalia of *Eriopis patagonia* Salazar, sp. nov. **—** Holotype. (**A**–**C**). Penis guide (pg), parameres (pm), phallobase (pb), and tegminal strut (ts) in lateral (**A**,**D**,**E**), dorsal (**B**), and ventral (**C**) views. (**D**,**E**). Details of the apical region of the pm and pg, and basal region of the ts showing the two small prolongations (arrowheads) and the deep concavity in the middle (arrow). cm: connective membrane 1 and 2. Dotted lines in (**A**–**C**) indicate membrane structures. Scale bars (mm): (**A**–**C**) 0.25; (**D**,**E**) 0.12.

Figure 1, Figure 2, Figure 3, Figure 4 and Figure 5.

**Type specimens. Holotype:** Patagonie [handwritten label]/*Eriopis connexa* Ger. v. *latepicta* Frm [handwritten label]/Muséum Paris 1930 Coll. Sicard/ (MNHN EC 10238 ♂). **Paratype:** *idem.* (MNHN EC 10239 ♂).

**Type locality**. Patagonia.

**Etymology.** The specific epithet refers to the only geographical locality known for the species.

**Diagnosis.***Eriopis patagonia* can be distinguished from the other *Eriopis* species that share the same geographical distribution by the following characters: body size smaller in *E. patagonia* (4.3 mm) and *Eriopis latepicta* Fairmaire (4.0 mm), and bigger in *Eriopis magellanica* (Philippi) (5.4 ± 0.4 mm, *n* = 20) and *Eriopis eschscholtzii* Mulsant (4.9 ± 0.3 mm, *n* = 20); *habitus* of the body in the lateral view less convex in *patagonia*, more convex in *eschscholtzii* and *magellanica*; matte black color integument in *patagonia* and shiny integument in *latepicta*, *eschscholtzii*, and *magellanica*; clypeus wider in *patagonia* and *eschscholtzii* and narrower in *magellanica*; body in general with less pubescence in *patagonia* than *magellanica* and *eschscholtzii*; eyes relatively less globular in *patagonia*, more so in *eschscholtzii* and *magellanica*; pronotum with different shape, relatively rounded oval in *patagonia*, oval in *magellanica,* and elongated oval in *eschscholtzii.* Lateral margins of the pronotum are less prominent in *patagonia* and more prominent in *eschscholtzii* and *magellanica*. Pronotal lateral “pits” narrow and shallow in *patagonia*, intermediate in following *eschscholtzii,* and relatively wider and deeper in *magellanica*; pronotum with a small yellow spot in the center of the anterior region in *patagonia* and *magellanica*, and with a complete yellow band joining the lateral spots in *latepicta* and *eschscholtzii*; the yellow spot of the lateral regions of the pronotum narrow and until the middle in *patagonia*, wider and extended along the lateral regions in *eschscholtzii* and *magellanica*, but in the latter interrupted in the middle; tibiae with few pubescence in *patagonia*, abundant setae in *eschscholtzii* and *magellanica*; elytra with larger punctuation and more abundant micropunctuation in *patagonia* than in *latepicta*, *eschscholtzii*, and *magellanica*; yellow spots on the discal region present in *magellanica* (including the darker morphs where they are slightly visible) and *eschscholtzii*, but not in the darker morph where the elytra are completely black except for a yellow band on the entire marginal edge of the elytra. These yellow spots in the discal region absent in *patagonia* and *latepicta*; *patagonia* is brachypterous and *eschscholtzii* and *magellanica* are macropterous; conformation of the male genitalia is different between *patagonia*, *eschscholtzii* and *magellanica* (data unknown for *patagonia* females and both sexes in *latepicta*).

**Description.***Habitus*: elongated and relatively flat (Figure 1A,B); body predominantly matte black, with small light yellow spots (color in dry specimens) in the dorsal and ventral part, in general body with relatively few pubescence and abundant micropunctuations and well-marked punctuations (Figure 1A,B and Figure 2A,B). Total length 4.3 mm (from labrum to posterior tip of the elytra), humeral width 1.4 mm, and at the middle of the elytra 2.01 mm in width.

***Head*** (Figure 1C,D and Figure 2A,C): 1.6 mm in length, 1.0 mm in width (between the eyes); dorsally with abundant micropunctuations, and large and deep punctuations (diameter 227 ± 2.7 µm, *n* = 10; distance between punctuations 241 ± 4.5 µm, *n* = 10), and scarce short setae. Labrum black with a yellow anterior border and with scarce short setae; anterior angles rounded and anterior margin almost straight (Figure 1C,D). Clypeus wide and yellow (Figure 1C,D and Figure 2A). Ocular canthus yellow in the middle and darker at the edges; anterior apex almost reaches the edge of the eyes (Figure 2A). Antennae brown light with scape (basal) short and wide, antennomere I (pedicel) shorter than II, II longer than III, and III shorter than IV (Figure 1E). Eyes not-so-globular (Figure 1A–D and Figure 2A). Ligule, menton, and submenton as in Figure 2F,G; ligula, maxillar, and labial palps brown light (Figure 2G).

***Thorax*** (Figure 1A–D, Figure 2D,G and Figure 3A): Pronotum oval (0.1 mm in length and 1.4 mm in width at the middle of the pronotum); anterior and posterior margins almost straight, lateral margins rounded, anterior angles rounded and almost level with the anterior margin; dorsal side with one yellow small triangular spot in the middle of the anterior region, a yellow spot almost square in the middle of the posterior region, and a yellow spot on the lateral regions from the anterior angle to the middle of the lateral region; pronotal lateral “pits” narrow and shallow (Figure 1A,B,D); thinly punctuate (punctuation diameter 84 ± 0.8 µm, *n* = 10; distance between punctuations 163 ± 1.0 µm, *n* = 10) (Figure 2D). Hypomeron black and narrow (Figure 2G and Figure 3A). Prosternum narrow, in general with scarce short setae but greater in number in the central region (Figure 2G and Figure 3A). Mesepisternum with some short setae to the margin near the mesosternum. Mesepimeron half black and half yellow. Metespisternum with short setae and one small yellow spot in the posterior angle (Figure 3A). Metepimeron narrow and yellow (Figure 3A). Mesosternum with few small punctuations and short setae (Figure 3A). Metaesternum with few and scattered short setae. Elytra (Figure 1A,B, Figure 2B,E, and Figure 3A) dorsally glabrous except the humeri with some short setae, with large and very well-marked punctuations (diameter 249 ± 2.7 µm, *n* = 10; distance between punctuations 257 ± 4.6 µm, *n* = 10) and other abundant micropunctuations between the punctuations; epipleura yellow with few short setae; each elytron with a light yellow spot starting from the basal region and continuing to the humeri-marginal region, stretched in the middle. Afterwards, the spot becomes narrower at the margin of the elytra, and near the apical region, it becomes wide again to form another spot, which afterwards narrows toward the apex of the elytra. Legs (Figure 1A,B,D and Figure 3A): femora with few and scattered short setae (dorsal view); tibiae with few short setae; protibiae measure 0.9 mm, mesotibiae 0.9 mm, metatibiae 1.13 mm in length respectively; tarsi brown. Hindwings reduced (brachypterous; Figure 3E).

***Abdomen***: Sternites with small punctuation and short setae; shape of the sternites as in Figure 3B,C. Tergites as in Figure 3D. Male Genitalia (Figure 4 and Figure 5): Penis 2.64 mm in length, tubular and well sclerotized; laterally punctuated and with amber-colored micro-spicules from the middle dorsal region to the distal region, before the apex (Figure 4B–D); apex of the penis, where the gonopore opens narrower and divided into three structures as in Figure 4D,E; the “penis capsule”, the most proximal region and most sclerotized part of the penis with two lateral extensions (inner and outer; Figure 4F). The outer extension (0.25 mm in length, 0.15 mm in width) divided into two asymmetrical parts, and the inner extension (0.19 mm in length, 0.21 mm in width) curved and forms a small concavity (0.1 mm in width). Both extensions with a well-marked curve in the base (lateral view; Figure 4F). Parameres (Figure 5A–D) short (0.54 mm in length) with abundant punctuations, well curved (lateral view), run almost in parallel and open slightly in the apical region (dorsal and ventral views); apex well rounded with abundant setae (Figure 5A–D); base strongly concave (dorsal view, Figure 5B). Phallobase (Figure 5A–C) elongated (0.45 mm in length, 0.15 mm in width, lateral view), almost the same size as the parameres and reaching almost half of the tegminal strut, with the ventral margin irregular and strongly sclerotized. Penis guide (phallus) (0.67 mm in length, 0.19 mm in width) with a deltoid shape and the base with a square shape (ventral view, Figure 5C); apex narrower, with abundant punctuations and almost at the level of the apex of the parameres (Figure 5D). Tegminal strut long (0.70 mm in length) and wide (0.21 mm in width), well sclerotized, deeply concave in the middle, and with two small prolongations in the base (Figure 5E).

**Variation in body color pattern.** The holotype specimen has one tiny spot over the spot that is near to the apical region of the right elytron (Figure 1B), but both spots overlap on the left elytron. There is also another small spot near the apex of the elytra (Figure 1B). The paratype does not have these additional spots. No other morphological variation was observed.

**Key characters.***Habitus* relatively flat; dorsal region of the body with matte black integument; pattern yellow of the elytra present in the humeri-marginal region, marginal region, and apical region, and absent in the discal region; elytra with abundant micropunctuations and large punctuations; pronotum rounded oval with small yellow spots; brachypterous.

**Distribution.** The label states that this species is distributed in South America, Patagonia Region, without any other specification.

**Nomenclatural remarks and comments.** We found these two specimens in Albert Sicard’s nominal collection at the MNHN. Sicard identified these specimens as *Eriopis connexa* Germar v. *latepicta* Fairmaire. The locality label and the identification label correspond to Sicard’s handwriting (Figure 1F). This identification label was found on one side of the specimens inside Sicard’s box, which agrees well with his style of organizing boxes. The specimens of *E. patagonia* do not have an exact collection date, and we have no other indication to infer a precise date. Sicard’s collection entered the MNHN in 1930. Thus, these specimens have as a minimum collection age of the date that the Sicard collection entered the MNHN; however, we can assume that the specimens were collected long before 1930. Another complication for dating the specimens is that other people’s collections of Coccinellidae entered the museum through Sicard [74].

**Next-generation sequencing output**. Of a total of 4,147,922 sequenced reads, 3,179,654 reads were retained after trimming the index library and the process of quality control. Of these, 27,612 corresponded to reads from the mitochondrial genome of this species. The mean coverage of the sequenced mitogenome was 142.8 (mean number of sequences covering a column in the assembly).

**Figure 6:**
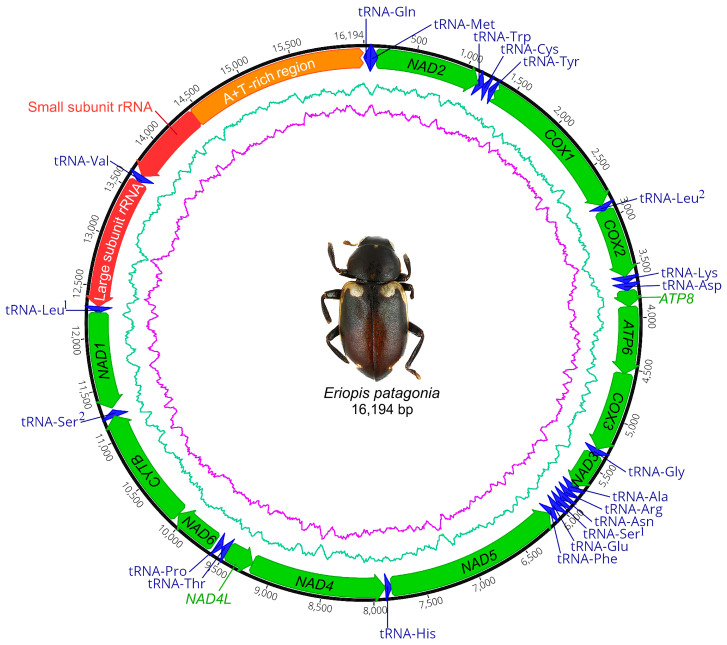
Map of the mitochondrial genome of *Eriopis patagonia* Salazar, sp. nov. The 13 protein-coding genes (PCGs) are shown in green, the 21 transfer RNA (tRNAs) are shown in blue, the two ribosomal RNA (rRNAs) are shown in red, and the A+T rich region is shown in orange. The direction of transcription is indicated by an arrow. Graphic representation of AT (green-blue) and GC (pink) content (%) and their changes throughout the mitogenome.

**Figure 7:**
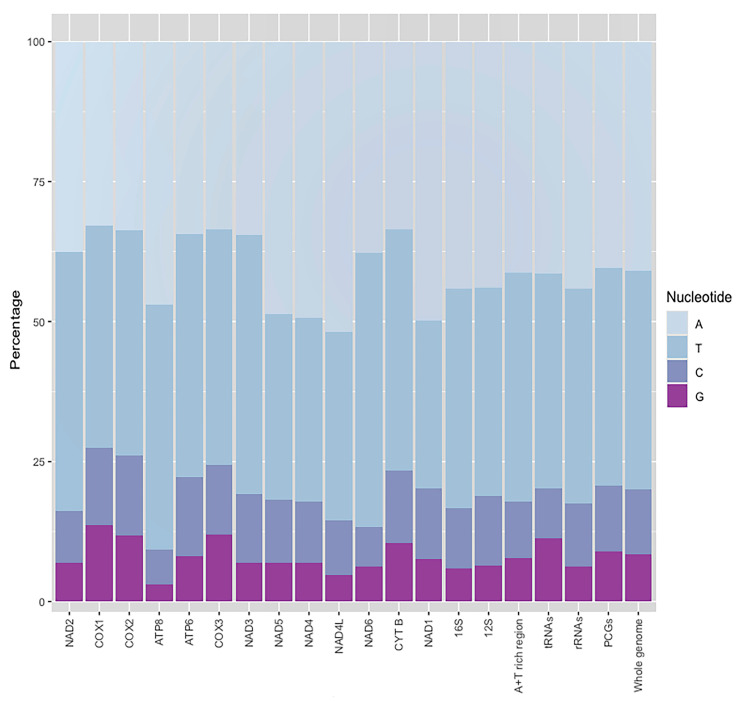
Nucleotide composition of the *Eriopis patagonia* mitogenome. Protein-coding genes (PCGs), transfer RNAs (tRNAs), and ribosomal RNAs (rRNAs).

**Figure 8:**
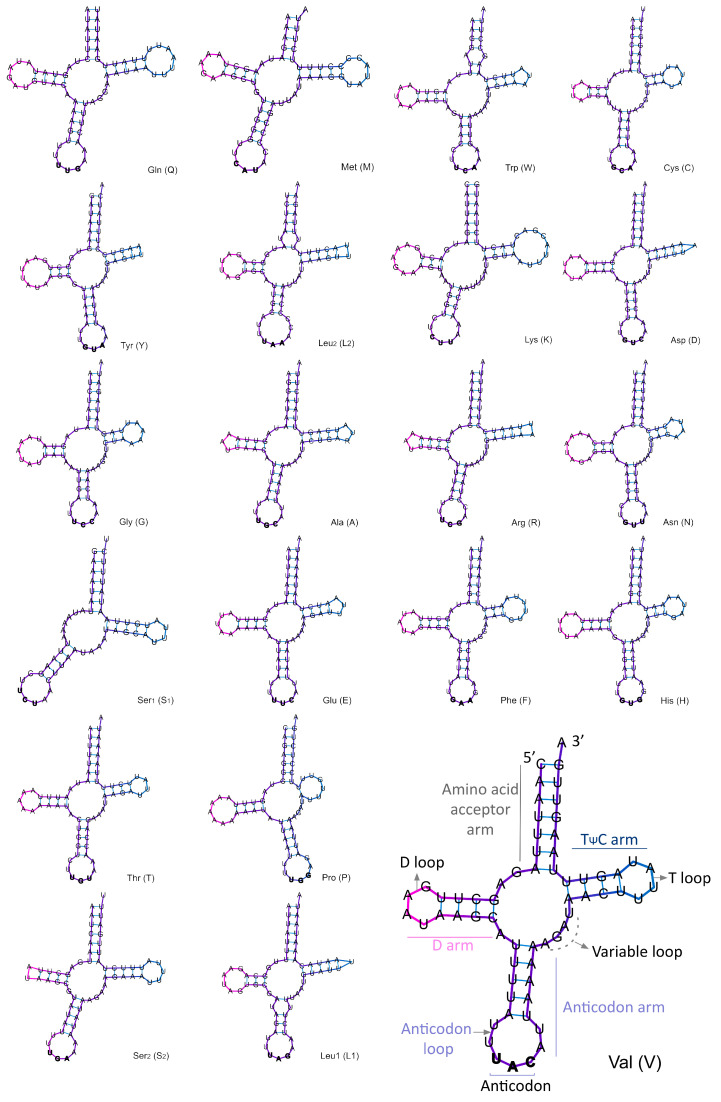
Predicted secondary structure of 21 transfer RNAs (tRNAs) of *Eriopis patagonia* mitogenome. Bars indicate Watson–Crick base pairings.

**Genome organization** (Table 2 and Table 3; Figure 6, Figure 7 and Figure 8). The circular mitogenome was determined to be 16,194 bp in length. This genome consists of 13 PCGs, two rRNA genes, a single non-coding A + T–rich region (1768 bp, the A + T ratio is 82.1%), and 21 tRNAs. Isoleucine tRNA (tRNA-Ile) was not detected due to incomplete sequencing. The 21 tRNAs have a range length from 60 to 70 bp and compose 1341 bp in total length. In regard to the secondary structure (Figure 8), all tRNA present a canonical cloverleaf secondary structure with the conventional four arms, except the tRNASer^1^, which lacks the D-arm and is replaced by a single loop. The tRNAs Try, Leu^2^, Asp, Arg, and Leu^1^ have a smaller T-loop motif. The tRNAs Cys, Ala, Arg, and Ser^2^ have a smaller D-loop motif. The general nucleotide composition of this genome is 20/79.9%, PCGs 20.8/79.2%, tRNAs 20.2/79.8%, and rRNAs 17.6/82.4% GC/AT ratio, respectively, with positive AT-skew and negative GC-skew (Table 3, Figure 7).

**Estimation of evolutionary divergence between mitogenome sequences** (Tables S2 and S3). The values resulting from the genetic divergence analysis between the paired mitochondrial sequences of Coccinelloidea species evaluated with PCGs vs. PCGs + rRNAs are similar. The ladybird beetle mitogenomes included in this study have a divergence range of ≈0.43–0.08 (number of base substitutions per site; Kimura 2-parameter; rate variation among sites modeled with a gamma distribution). The highest calculated divergence value was between *Henosepilachna pusillanima* and *Coleomegilla maculata,* and the lowest was between *E. patagonia* and *E. connexa*. These *Eriopis* species have the least divergent mitogenomes compared to other taxa within a same genus (two representatives of *Calvia* Mulsant 0.17; *Coccinella* Linnaeus 0.13; *Cycloneda* Crotch 0.16; *Harmonia* Mulsant 0.25; *Henosepilachna* Li and Cook 0.20; *Hippodamia* Dejean 0.22; *Propylea* Mulsant 0.11, respectively). The two outgroups *Dastarcus helophoroides* and *Gloesoma* sp. have the highest divergence sequence values in comparison to the Coccinellidae.

**Figure 9:**
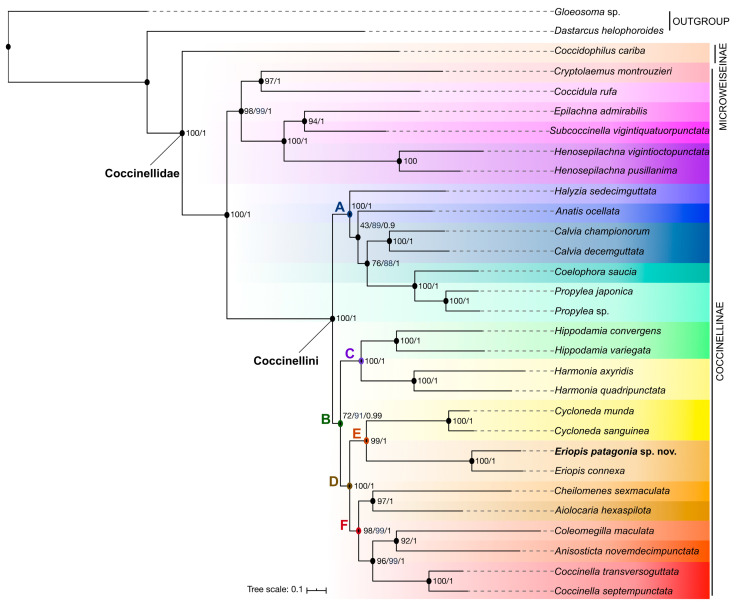
Phylogenetic relationships of Coccinellidae. Tree inferred by the maximum likelihood estimation method (ML) based on 13 protein-coding genes (including all codon positions) and two ribosomal RNAs from mitochondrial genomes of 28 Coccinellidae and two out-groups. ML bootstrap support and posterior probability values are indicated at the right of each node. Transfer bootstrap expectation value (blue) is only shown when it differs from the bootstrap value.

**Phylogenetic analysis** (Figure 9 and Figures S3–S5). The phylogenetic analysis of the 15 mitochondrial markers from 27 additional Coccinellidae species confirms that the sequenced specimen of *E. patagonia* belongs to the *Eriopis* genus within the Coccinellini tribe and is sister species to *E. connexa*. *Eriopis* is the sister group of *Cycloneda* (clade E). These results were recovered in all partitioned schemes using ML and BI analyses.

In regard to the phylogeny of Coccinellidae, both BI and ML analyses based on 13 mitochondrial protein-coding genes (including all codon positions), 12S rRNA and 16S rRNA (partition PCG_RNA) obtain the same topology (Figure 9 and Figure S3). Most of the clades in these phylogenetic inferences are supported by high support values (FB >70; TBE >88; PP >0.99, respectively), with exception of the clade (*Anatis* Mulsant *+* ((*Coelophora* + *Propylea*) + *Calvia*)) (FB= 43; TBE = 89; PP = 0.9). The phylogenetic trees recovered from the concatenated datasets with the two first partitioning strategies (PCG_RNA, PCG12_RNA) and inferred using ML and BI methods showed consistent topologies and similar nodal support values (Figure 9 and Figure S4). The clade (*Anatis +* ((*Coelophora* + *Propylea*) + *Calvia*)) has higher support (FB = 90; TBE = 98; BB = 1) in the second partitioning strategy (Figure S4). The third partition scheme (PCGs_AA, translated into amino acids; Figure S5) produced a variant of the ML tree topology with *Anatis* found to be sister to *Halyzia* Mulsant, with a moderate support value (FB = 70; PP = 0.92), and the clade *Cryptolaemus* Mulsant + *Coccidula* Kugelann was not recovered as sister of the Epilachnini clade. The basal node of the D and F clades has relatively lower support values (FB = 71; 48, respectively), and the basal node of the B clade has a higher support (FB = 90). In addition, the BI analysis from this partition scheme does not recovery several of the clades.

## 4. Discussion

We combined museum genomic and morphological studies to describe a new species of ladybird beetle. With the discovery of the only two known specimens of *E. patagonia*, we were able to use non-destructive molecular techniques and next-generation sequencing to characterize its mitochondrial genome and infer its phylogenetic position.

### 4.1. Morphological Considerations and Geographical Distribution

*Eriopis patagonia* is morphologically very close to *E. latepicta*. Based on the brief description of *E. latepicta* by Fairmaire [75], the most relevant characters that differentiate these species are the *habitus* of the body (flat vs. convex), punctuation of the elytra, integument brightness and the extension of the light spot on the anterior region of the pronotum. *Eriopis patagonia* has only a tiny spot in the central part of the anterior region of the pronotum, while in *E. latepicta*, a yellow band completely covers the anterior region and joins with the lateral spots of the pronotum. This feature of the pronotum can be polymorphic or not in other *Eriopis* species. For example, all morphotypes of *E. eschscholtzii* have an entire yellow band on the anterior region joining with the lateral spots of the pronotum. *Eriopis concordia* González has an entire band on the anterior region of the pronotum that continues into the lateral region; however, some specimens only have a spot in the central part of the anterior region that does not join with the lateral spots. In *Eriopis loaensis* González, the situation is opposite to that of *E. concordia*, whereby most specimens have only one spot on the anterior region of the pronotum and a few have a yellow band [36].

A key character distinguishing *E. latepicta* and *E. patagonia* is the punctuation of the elytra, which generally does not vary intraspecifically. The former has very small punctuations, whereas the latter has larger and marked punctuation and abundant micropunctuation. In addition, Fairmaire [75] mentioned that *E. latepicta* has a shiny black color and that it could have (but he did not affirm this) two spots on the discal region of the elytra. The new species has a matte black color and has no clear spots in the discal region of the elytra. These two discal spots are generally common in most species of *Eriopis*. Some Peruvian species (*Eriopis santiagoi* Bustamante & Oroz, *Eriopis lawalawani* Bustamante, González & Oroz) with a predominance of black coloring in the elytra have one discal spot in each elytron [76]. In the light of the description of *E. latepicta*, this species and *E. patagonia* share almost the same elytra coloration pattern. Unfortunately, we cannot compare its other morphological characters with those of *E. patagonia* because neither we nor anybody else have yet located the type series of *E. latepicta* studied by Fairmaire. *Eriopis latepicta* is a species that continues to have an uncertain taxonomic status.

The type locality of *E. patagonia* is Patagonia, which is a wide region between Argentina and Chile in the south of South America. In the same way, Patagonia is mentioned as the type locality of *E. latepicta* [75]. This is one of the reasons why we believe that Sicard identified the specimens of the new species as *E. latepicta*. We suggest that *Eriopis patagonia* was probably collected somewhere in western Patagonia toward the Andes, because it is a brachypterous species. Increases of certain factors (e.g., wind, cold, geographical isolation) at high altitude have been correlated with wing reduction in other Coleoptera (e.g., Carabidae reviewed by [77]; Passalidae [78]). Thus, it is unlikely that *E. patagonia* was collected from flat areas in the eastern part of this region.

Two other species of *Eriopis* have been reported in Patagonia. *Eriopis eschscholtzii* and *E. magellanica* are morphologically distinct from *E. patagonia*. These two species, in addition to the typical black coloration patterns with yellow spots, include specimens with predominantly black coloration, which could be confused with *E. patagonia*. The “darkest” morph of *E. eschscholtzii* has completely black elytra, except for the presence of a yellow band along the margin of the elytra. The most common morphotypes of *E. eschscholtzii* have elytra with large yellow spots, which are sometimes joined together as a band and where the black is reduced [36,79]. The “darkest” morph of *E. magellanica* has very reduced yellow spots, but these are slightly visible in the discal region of the elytra. In this study, we included other external characteristics of the body besides color pattern, which are useful for distinguishing these species. In regard to the distribution, the darkest morphs of *E. eschscholtzii* have been reported in the Zona Austral of Chile [36]. In this geographical zone, a specimen was reported in El Valle del Lago Blanco, Río Senguer Department, Chubut Province, Argentina [80] and another was discovered in the XI Region, Balmaceda collected in 1999 and deposited in the personal collection of Manuel Diéguez, Santiago de Chile (http://www.coccinellidae.cl, accessed in 16 June 2018). The darkest morphs of *E. magellanica* have been reported in Tierra del Fuego, Chile (material observed by us) and Tierra del Fuego, Gallegos Chico, Última Esperanza, Parque Nacional Torres del Paine, Chile (http://www.coccinellidae.cl, accessed in 16 June 2018).

### 4.2. Genome Organization

We sequenced and assembled the mitogenome of one historical and nomenclatural type specimen of *E. patagonia*. It is the first published and characterized mitogenome for this genus obtained from an old specimen from a Natural History Collection. The order and orientation of the genes in the mitogenome of *E. patagonia* are consistent with those of other mitochondrial genomes of Coccinellidae [42,62,63,64,65,66,67,68,69,70,71,72,73] and contain the typical set of mitochondrial genes found in insects.

The predicted secondary structure and anticodon sequence for 21 tRNAs of the *E. patagonia* mitogenome is similar that reported to other ladybird beetles [65,71]. As was also noted by [65], the most variations are in substitutions, and indels (insertion, deletion of bases) of the tRNA among the Coccinellidae are present in the variable and D loops, and TψC arm, which is reflected in the differences in the size of this arm and its D-loop. *Eriopis patagonia* share with *Aiolocaria hexaspilota*, *Calvia muiri* (Timberlake), *C. septempunctata*, *Illeis cincta* Fabricius, and *Propylea japonica* a lack of the TψC arm in tRNA-Pro, which is replaced by the loop [71], while in other species, the TψC arm is present in this tRNA [65]. The *Eriopis patagonia* mitogenome has the smallest D-loop in tRNA-Try and tRNA-Leu^2^ in comparison with the other studied ladybird beetles where the secondary structure of mitochondrial tRNAs was predicted.

Regarding the species of this genus, *E. patagonia* and *E. connexa* (GenBank section number MG253268; unpublished mitogenome) share high similarity in the organization and genetic composition of their mitochondrial genomes (Figures S1 and S2B). Furthermore, both sequenced mitogenomes have the lowest genetic divergence (compared to the divergence values between all taxa of ladybird beetles and species of a same genus analyzed here; Tables S2 and S3). The differences were sequence length (*E. patagonia* 16,194 bp vs. *E. connexa* 17,652 bp in length) and nucleotide composition in whole mitogenome, PCGs, tRNAs, and rRNAs (see Figure 7, Table 3 to *E. patagonia* vs. *E. connexa*: 20.6/79.5; 21.2/78.8; 21.6/78.4; 17.8/82.3% GC/AT ratio, respectively; Figure S2B).

The secondary structure and composition of nucleotides of the most mitochondrial tRNAs are highly conserved between both *Eriopis* species. We noted differences in the sequences and structures of T and D loops in some tRNAs (Asp, Arg, Asn, Glu, Thr, Pro) (Figure 8 and Figure S2A). Based on the prediction by MITOS, particularly the tRNA-Gln in *E. connexa* mitogenome differs in the sequence of the amino acid acceptor arm and the lack of the TψC arm and typical T-loop (Figure S2A). The latter could be evaluated with other bioinformatics tools available for identifying mitochondrial tRNAs. Although there are exceptions, the most typical in almost all metazoic mitochondrial tRNAs is to have a canonical structure of clover leaves, save for the missing D-arm in the tRNA-Ser [81,82].

The *E. connexa* mitogenome has an A+T-rich region of 1747 bp in length with 13.8/86.2% GC/AT ratio. Downstream of this region is tRNA-Ile, which is separated from tRNA-Gln by a 1382 bp-long intergenic spacer. Sequencing failed to recover this intergenic spacer and tRNA-Ile in *E. patagonia*; however, most if not the entire control region was recovered (1768 bp; 17.9/82.1% GC/AT ratio). This intergenic spacer, which varies in length, is present in other ladybird beetles [71] and could be present in the mitogenome of other *Eriopis* species.

The size of the *E. patagonia* mitogenome is similar to that of other Coccinellini (see Table 1; e.g., *Anisosticta novemdecimpunctata*, *Calvia decemguttata*, *Coccinella transversoguttata*, *Halyzia sedecimguttata*, and *P. japonica*). All mitochondrial genomes of these Coccinellini were determined to be partial mitogenomes because that tRNA-Ile (Isoleucine) was not recovered from the genomic library. The largest complete mitogenome in Coccinellidae was reported in *C. septempunctata* with a total length of 18,965 bp, which includes a larger control region of 4469 bp [42]. Other species with complete mitochondrial genomes have a relatively short control region—for example, the *A. hexaspilota* mitogenome with a total length of 17,549 bp and 1603 bp corresponding to the A+T-rich region [62], *Harmonia quadripunctata* with 18,051 bp and 2071 bp (unpublished), and *Hippodamia variegata* 17,823 bp and 1590 bp [68], respectively. In general, differences in the length of the control region and the lack of the first transfer RNA in the assembled mitogenomes may be due to the fact that this region has a low recovery.

### 4.3. Phylogenetic Considerations

Our phylogenetic analysis based on mitochondrial genomes (13 PCGs + two rRNAs) from 28 Coccinellidae recovered two main clades (Coccinellinae and Microweiseinae). Coccinellinae is also divided into two main clades (clade Coccinellini and the other clade including Coccidulini, Epilachnini, Scymnini) with high support (PCG_RNA and PCG12_RNA: FB and TBE = 100; PP = 1.0 / PCG_AA: FB = 67; PP = 0.84). The generic composition between the main clades and inside each clade of Coccinellini (here A–F) differs slightly to the previous studies of Escalona et al. [83], which includes one mitochondrial and four nuclear markers that Song et al. [71] and Yuan et al. [65] based on complete mitochondrial genomes. Regarding the main clades within Coccinellini, here, this tribe is divided into two principal clades (A and B, the latter including clades C and D), as also recovered by Song et al. ([71]; Figure 3 and Figure 4). Contrarily, in Escalona et al. [83], the clade “1” (where is the clade E) was recovered as the early-diverging clade of Coccinellini and being sister to the clade that includes the clades “2” and “3”. The clade “3” is split in two clades (here A and C) that are closely related, while in our analysis, the clade C was sister to clade D. Here, clade D comprises clades “1” and “2” of Escalona et al. [83]. In Song et al. ([71]; Figure S5), the close relationship between clades A and C was recovered when the gene dataset includes 13 PCGs excluding the third-codon positions combined with the 24 RNA genes using ML analysis.

The phylogenetic relationships between the genera within clade A were almost the same as in Song et al. [71], except for the position of *Epilachna* Chevrolat, which was found to be sister to *Subcoccinella* Agassiz. The only difference with the results of Escalona et al. [83] is that *Anatis* and *Halyzia* were not sister genera. However, both genera were recovered to be sister when our phylogenetic analysis includes only the mitochondrial PCGs that translate into amino acids. The close relationship between *Hippodamia* and *Harmonia* (clade C) was also recovered in previous studies [71,83].

Our analysis also confirmed the close phylogenetic relationship between the South American genera *Eriopis* and *Cycloneda* (clade E). The latter has a wider distribution than *Eriopis*, and it is found from North to South America, including the Caribbean [84]. This result has already been proposed by ([71]; Figure 4) and [83]. However, in the first study, this relationship was not recovered when the dataset included PCGs with all codon positions combined with the 24 RNA genes ([71] Figure 3).

In clade F, we found *Cheilomenes* Chevrolat as sister to *Aiolocaria* (Hope), whereas *Cheilomenes* was found to be more closely related to *Anisosticta* Chevrolat in previous studies [65,71]. In agreement with Escalona et al. [83], we found *Coleomegilla* Timberlake to be sister to *Anisosticta*. In our study, *Coccinella* was nested within clade F, as also proposed by Song et al. ([71]; Figure 4 and Figure S5). However, here, *Coccinella* was recovered as the sister group to the clade *Coleomegilla* and *Anisosticta*, while in previous studies, this genus was found to be sister group to all other genera in clade F [83] or as sister to *Cycloneda* ([71]; Figure 3).

These differences in the phylogenetic hypotheses of ladybird beetles discussed here could be due to the influence of the heterogeneity in nucleotide composition in the molecular dataset. Song et al. [71] indicated that the mitochondrial genes of ladybird beetles have significant saturation in the third codon position of the 13 PCGs and when PCGs are combined with the RNAs (two rRNA and 22 tRNA). We observed only two differences in the backbone of the tree and a slight variation in node support in our analyses under the amino acid partitioning scheme. Removing the third codon position of the mitochondrial PCGs in our datasets did not have an effect on the topology of the tree and the support values. The different resulting topologies and/or the support values of the nodes also could be the influence of the evolutionary substitution models (homogeneous vs. non-homogeneous), the data partition strategy (PCGs: partitioned by gene vs. gene and codon position; including all codon positions or excluding the third codon position), and the molecular markers (nuclear genes/mitochondrial: PCGs, rRNAs, tRNAs/combination of them) used in the phylogenetic inferences, which change in each study compared here. Other studies have supported that nucleoid heterogeneity, specially in mitochondrial gens, data partitioning, and evolutionary model selection can significantly influence the results of phylogenetic analysis (e.g., [85,86,87]). A broader data exploration taking into account the influence of the mentioned factors could be interesting to infer a new phylogeny of Coccinellidae with a larger sample of taxa and molecular markers.

## 5. Conclusions

The combination of morphology and museum collection genomics has allowed us to discover a new species of ladybird beetle, *Eriopis patagonia*, and infer its phylogenetic relationship based on the mitochondrial genome. We stress the importance of Natural History Collections as a source of genetic information, since NGS technology and genome skimming methods are very useful for obtaining molecular information from old museum specimens. For many ladybird beetle genera, especially those distributed in South America, genetic information is still lacking. Considering that in certain cases, it is not easy to obtain fresh specimens due to the difficulties in doing fieldwork (e.g., obtaining collection permits, costs, difficulty of access in some geographical areas), NGS is an attractive tool for continuing the molecular systematic study of Coccinellidae using material from biological collections.

## Figures and Tables

**Table 1:** Species of Coccinelloidea used in the phylogenetic analysis. Respective GenBank accession numbers of the mitochondrial genomes and references are given.

Family Subfamily	Species	Author	Mitogenome			Reference
			Length (bp)	Partial—Complete +	GenBank Code	
Corylophidae						
Corylophinae	*Gloeosoma* sp.		12,474	–	JX412843	Unpublished
Bothrideridae						
Bothriderinae	*Dastarcus helophoroides*	(Fairmaire)	15,878	+	NC_024271	[61]
Coccinellidae						
Coccinellinae	*Aiolocaria hexaspilota*	(Hope)	17,549	+	MK583344	[62]
	*Anatis ocellata*	(Linnaeus)	17,092	+	NC_036272	Unpublished
	*Anisosticta novemdecimpunctata*	(Linnaeus)	15,289	–	KT876880	[63]
	*Calvia champinorum*	Booth	17,575	–	KX132085	Unpublished
	*Calvia decemguttata*	(Linnaeus)	16,425	–	KX087252	Unpublished
	*Cheilomenes sexmaculata*	Fabricius	17,192	–	KM244706	[64]
	*Coccinella septempunctata*	Linnaeus	18,965	+	JQ321839	[42]
	*Coccinella transversoguttata*	Faldermann	15,806	–	MG584726	[65]
	*Coleomegilla maculata*	De Geer	17,516	–	KJ778881	Unpublished
	*Cycloneda munda*	(Say)	14,292	–	KJ778882	Unpublished
	*Cycloneda sanguinea*	(Linnaeus)	15,137	+ *	KU877170	[66]
	*Eriopis connexa*	(Germar)	17,652	+	MG253268	Unpublished
	***Eriopis patagonia***	**Salazar**	**16,194**	**–**	**MN509443**	**This study**
	*Halyzia sedecimguttata*	(Linnaeus)	15,766	–	KT780652	Unpublished
	*Harmonia axyridis*	(Pallas)	16,382	–	KR108208	[67]
	*Harmonia quadripunctata*	(Pontoppidan)	18,051	+ *	KX087296	Unpublished
	*Hippodamia convergens*	Guérin-Méneville	18,419	+	KX755331	[66]
	*Hippodamia variegata*	(Goeze)	17,823	+	MK334129	[68]
	*Coelophora saucia*	(Mulsant)	14,106	–	MK574678	[69]
	*Propylea japonica*	(Thunberg)	15,027	–	KM244660	[64]
	*Propylea* sp.		15,915	–	KX132084	Unpublished
	*Henosepilachna pusillanima*	(Mulsant)	16,216	+	NC_023469	[70]
	*Henosepilacna vigintioctopunctata*	(Fabricius)	17,057	+	NC_041172	Unpublished
	*Epilachna admirabilis*	Crotch	17,445	+ *	MN053053	[71]
	*Subcoccinella vigintiquatuorpunctata*	(Linnaeus)	14,645	–	KT780695	Unpublished
	*Coccidula rufa*	Herbst	10,589	–	JX412767	Unpublished
	*Cryptolaemus montrouzieri*	Mulsant	17,010	+	KT874575	[72]
Microweiseinae	*Coccidophilus cariba*	Gordon	15,343	+	MN447521	[73]

* Complete mitogenome after our verification of the gene notation.

**Table 2:** Summary of *Eriopis patagonia* mitogenome annotation. The exponent numerals in gene column are used to differentiate each of the two Leucine- and Serine-specifying transfer RNAs (tRNAs) (Leu^1^ and Leu^2^, Ser^1^ and Ser^2^); TAA^1^ stop codon is completed by the addition of 3′A residues to mRNA; (–) stop codon not determined.

Gene	Location	Length (bp)	Codon		Anticodon		Strand Reverse – Forward +
			Start	Stop	Sequence	Location	
tRNA-Ile	?	?				?	
tRNA-Gln	1–70	70			TTG	37–39	–
tRNA-Met	68–136	69			CAT	98–100	+
*NAD2*	133–1143	1011	ATA	TAA			+
tRNA-Trp	1141–1204	64			TCA	1172–1174	+
tRNA-Cys	1197–1258	62			GCA	1227–1229	–
tRNA-Tyr	1261–1323	63			GTA	1291–1293	–
*COX1*	<1326–2864	>1539	AAT	TAA			+
tRNA-Leu^2^	2862–2921	60			TAA	2889–2891	+
*COX2*	2923–3601	679	ATA	TAA^1^			+
tRNA-Lys	3602–3670	69			CTT	3632–3634	+
tRNA-Asp	3671–3734	64			GTC	3702–3704	+
*ATP8*	3735–3896	162	ATA	TAA			+
*ATP6*	3890–4546	657	ATG	TAA			+
*COX3*	4549–5329	781	ATG	TAA^1^			+
tRNA-Gly	5330–5392	63			TCC	5360–5362	+
*NAD3*	5390–5746	357	ATA	TAG			+
tRNA-Ala	5743–5807	65			TGC	5773–5775	+
tRNA-Arg	5806–5869	64			TCG	5835–5837	+
tRNA-Asn	5866–5930	65			GTT	5898–5900	+
tRNA-Ser^1^	5931–5986	56			TCT	5951–5953	+
tRNA-Glu	5986–6049	64			TTC	6017–6019	+
tRNA-Phe	6048–6111	64			GAA	6079–6081	–
*NAD5*	6111–7828	1718	TAT	–			–
tRNA-His	7826–7889	64			GTG	7856–7858	–
*NAD4*	7889–9205	1317	TAT	TTA			–
*NAD4L*	9205–9480	276	TAT	TTA			–
tRNA-Thr	9482–9545	64			TGT	9512–9514	+
tRNA-Pro	9546–9607	62			TGG	9573–9575	–
*NAD6*	9637–10,079	443	ATA	–			+
*CYT B*	10,079–11,221	1143	ATG	TAA			+
tRNA-Ser^2^	11,220–11,283	64			TGA	11,247–11,249	+
*NAD1*	11,301–12,245	945	TAT	CTA			–
tRNA-Leu^1^	12,243–12,304	62			TAG	12,273–12,275	–
*Large subunit rRNA*	12,305–13,580	1284					–
tRNA-Val	13,590–13,652	63			TAC	13,621–13,623	–
*Small subunit rRNA*	13,651–14,426	776					–
A + T rich region	14,427– >16,194	>1768					

**Table 3:** Nucleotide composition of the *Eriopis patagonia* mitogenome. Protein-coding genes (PCGs), transfer RNAs (tRNAs), and ribosomal RNAs (rRNAs).

Feature	Proportion (%)						Skews		N° of Nucleotides (bp)
	A	T	A+T	G	C	G+C	AT	GC	
Whole genome	41.0	38.9	79.9	8.5	11.6	20.0	0.03	−0.15	16,194
PCGs	40.4	38.8	79.2	8.9	11.9	20.8	0.02	−0.14	11,028
*NAD2*	37.6	46.2	83.8	6.9	9.3	16.2	−0.10	−0.15	
*COX1*	32.9	39.6	72.5	13.6	13.9	27.5	−0.09	−0.01	
*COX2*	33.7	40.2	73.9	11.8	14.3	26.1	−0.09	−0.1	
*ATP8*	46.9	43.8	90.7	3.1	6.2	9.3	0.03	−0.33	
*ATP6*	34.4	43.4	77.8	8.2	14.0	22.2	−0.12	−0.26	
*COX3*	33.5	41.9	75.4	12.0	12.5	24.5	−0.11	−0.02	
*NAD3*	34.5	46.2	80.7	7.0	12.3	19.3	−0.14	−0.27	
*NAD5*	48.5	33.2	81.7	7.0	11.2	18.2	0.19	−0.23	
*NAD4*	49.4	32.8	82.2	6.9	10.9	17.8	0.20	−0.22	
*NAD4L*	51.8	33.7	85.5	4.7	9.8	14.5	0.21	−0.35	
*NAD6*	37.7	49.0	86.7	6.3	7.0	13.3	−0.13	−0.05	
*CYT B*	33.6	42.9	76.5	10.5	13.0	23.5	−0.12	−0.11	
*NAD1*	49.8	29.9	79.7	7.6	12.6	20.2	0.25	−0.25	
*Large subunit rRNA*	44.2	39.1	83.3	6.0	10.7	16.7	0.06	−0.28	
*Small subunit rRNA*	43.9	37.1	81.0	6.4	12.5	18.9	0.08	−0.32	
A+T rich region	41.3	40.8	82.1	7.8	10.1	17.9	0.01	−0.13	
*tRNAs*	41.4	38.4	79.8	8.9	11.3	20.2	0.04	−0.12	1341
*rRNAs*	44.1	38.3	82.4	6.2	11.4	17.6	0.07	−0.3	2060

## Data Availability

The sequenced mitogenome is available in GenBank under accession number MN509443. The nucleotide multiple sequence alignment used for the phylogenetic analyses are available in Mendely Dataset (Mendeley Data, V1, doi:10.17632/fn8gh9kcpc.1).

## Supplementary Materials

The following are available online at https://www.mdpi.com/2075-4450/11/11/766/s1. Table S1. The partition schemes and best substitution models proposed by Partition Finder. Table S2. Estimates of evolutionary divergence between pairwise mitochondrial sequences of Coccinelloidea species. The value is the number of base substitutions per site between nucleotide sequences estimated by the Kimura-2-parameter model, and the variation rate among sites was modeled with a gamma distribution. The analysis was based on 9122 positions that comprise the two rRNAs and PCGs with the three-codon positions for 30 taxa. The out-groups (in blue) are included to compare with the in-group (Coccinellidae). Table S3. Estimates of evolutionary divergence between pairwise mitochondrial sequences of Coccinelloidea species. The value is the number of base substitutions per site between nucleotide sequences estimated by the Kimura-2-parameter model, and the variation rate among sites was modeled with a gamma distribution. The analysis was based on 9452 positions that comprise the PCGs with the three-codon positions for 30 taxa. The out-groups (in blue) are included to compare with the in-group (Coccinellidae). Figure S1. Maps of *Eriopis patagonia* Salazar and *Eriopis connexa* (Germar) mitogenomes. PCGs are shown in green, tRNAs are shown in blue, rRNAs genes are shown in red, and the A+T rich region is shown in orange. The direction of transcription is indicated by an arrow. The tRNAs are labeled according to the IUPAC-IUB single letter amino acid code. The dotted lines in *E. patagonia* mitogenome indicate that the intergenic spacer and tRNA-I were not recovered in this study. Figure S2. *Eriopis connexa* mitogenome. (**A**). Predicted secondary structure of transfer RNAs (tRNAs). The tRNAs are labeled according to the IUPAC-IUB amino acid code. Orange circles represent changes in the sequence with respect to tRNAs of the *E. patagonia* mitogenome. Bars indicate Watson–Crick base pairings. (**B**). Nucleotide composition. Protein-coding genes (PCGs), tRNAs, and ribosomal RNAs (rRNAs). Figure S3. Phylogenetic relationships of Coccinellidae. Tree inferred by the Bayesian Inference (BI) method based on 13 protein-coding genes including all codon positions and two ribosomal RNAs (PCG_RNA) from mitochondrial genomes of 28 Coccinellidae and two out-groups. Bayesian posterior probability values are indicated at the right of each node. Figure S4. Phylogenetic relationships of Coccinellidae. Tree inferred by the maximum likelihood estimation method (ML) based on 13 protein-coding genes excluding the third-codon positions and two ribosomal RNAs (PCG12_RNA) from mitochondrial genomes of 28 Coccinellidae and two out-groups. ML bootstrap support values and posterior probability values are indicated at the right of each node. Transfer bootstrap expectation value (blue) is only shown when it differs from the bootstrap value. Figure S5. Phylogenetic relationships of Coccinellidae. Tree inferred by the maximum likelihood estimation method (ML) based on 13 protein-coding genes being translated into amino acids (PCG_AA) from mitochondrial genomes of 28 Coccinellidae and two outgroups. ML bootstrap support values and posterior probability values are indicated at the right of each node. The abbreviation ‘‘- -” indicates that the node is not recovered by the BI analysis.

## New Species Registration

The following information notes the registration of the new species: Publication LSID: urn:lsid:zoobank.org:pub:4429ABB0-9247-4C6B-B625-0B66619AA06F//*Eriopis patagonia* Salazar sp. nov. LSID: urn:lsid:zoobank.org:act:AADAA2C8-01CF-4EB6-A0DA-20EA885D88F7.
