# Supplementary material for: New Light on Historical Specimens Reveals a New Species of Ladybird (Coleoptera: Coccinellidae): Morphological, Museomic, and Phylogenetic Analyses
Source: Insects. 2020 Nov 6;11(11):766. doi: 10.3390/insects11110766 (PMC7694756; doi:10.3390/insects11110766)
Supplement: Supplementary file 1 [file insects-11-00766-s001.zip › Supplementary_files_FINAL-VERSION_970082/Table S2__FINAL-VERSION_970082.pdf]

Table S2. Estimates of evolutionary divergence between pairwise mitochondrial sequences of Coccinelloidea species.

The value is the number of base substitutions per site between nucleotide sequences estimated by the Kimura-2-parameter model, and the variation rate among sites was modeled with a gamma distribution. The analysis was based on 9122 positions that comprise the two rRNAs and PCGs with the three-codon positions for 30 taxa. The out-groups (in blue) are included for comparision with the in-group (Coccinellidae).

**Species names**	*Aiolocaria hexaspilota*	*Anatis ocellata*	*Anisosticta novemdecimpunctata*	*Calvia championorum*	*Calvia decemguttata*	*Cheilomenes sexmaculata*	*Coccidophilus cariba*	*Coccidula rufa* **	*Coccinella septempunctata*	*Coccinella transversoguttata*	*Coleomegilla maculata*	*Cryptolaemus montrouzieri*	*Cycloneda munda* *	*Cycloneda sanguinea*	*Epilachna admirabilis*	*Eriopis connexa*	***Eriopis patagonia***	*Halyzia sedecimguttata*	*Harmonia axyridis*	*Harmonia quadripunctata*	*Henosepilachna pusillanima*	*Henosepilachna vigintioctopunctata*	*Hippodamia convergens*	*Hippodamia variegata*	*Lemnia saucia*	*Propylea japonica*	*Propylea* sp.	*Subcoccinella vigintiquatuorpunctata* **	*Dastarcus helophoroides*
Anatis ocellata	0.215																												
Anisosticta novemdecimpunctata	0.245	0.274																											
Calvia championorum	0.227	0.192	0.268																										
Calvia decemguttata	0.229	0.191	0.275	**0.170**																									
Cheilomenes sexmaculata	0.230	0.256	0.277	0.264	0.260																								
Coccidophilus cariba	0.359	0.360	0.372	0.366	0.365	0.384																							
Coccidula rufa **	0.340	0.343	0.369	0.341	0.347	0.376	0.358																						
Coccinella septempunctata	0.214	0.232	0.254	0.235	0.233	0.257	0.363	0.366																					
Coccinella transversoguttata	0.218	0.237	0.267	0.243	0.240	0.258	0.377	0.370	**0.127**																				
Coleomegilla maculata	0.291	0.307	0.303	0.301	0.315	0.311	0.412	0.413	0.260	0.277																			
Cryptolaemus montrouzieri	0.323	0.334	0.359	0.346	0.343	0.355	0.355	0.333	0.348	0.353	0.385																		
Cycloneda munda *	0.260	0.274	0.306	0.285	0.280	0.299	0.393	0.399	0.283	0.283	0.334	0.389																	
Cycloneda sanguinea	0.261	0.281	0.307	0.288	0.286	0.304	0.393	0.404	0.278	0.289	0.341	0.391	**0.160**																
Epilachna admirabilis	0.290	0.306	0.324	0.311	0.314	0.336	0.311	0.292	0.320	0.331	0.363	0.286	0.363	0.360															
Eriopis connexa	0.214	0.223	0.255	0.233	0.239	0.250	0.362	0.354	0.223	0.233	0.296	0.337	0.266	0.276	0.306														
Eriopis patagonia	0.209	0.226	0.257	0.236	0.236	0.255	0.366	0.355	0.225	0.231	0.296	0.331	0.263	0.273	0.305	**0.085**													
Halyzia sedecimguttata	0.233	0.203	0.279	0.223	0.220	0.269	0.377	0.350	0.245	0.250	0.324	0.351	0.289	0.299	0.314	0.247	0.249												
Harmonia axyridis	0.264	0.272	0.303	0.275	0.284	0.300	0.393	0.394	0.277	0.273	0.340	0.373	0.319	0.318	0.351	0.286	0.279	0.285											
Harmonia quadripunctata	0.273	0.273	0.306	0.288	0.286	0.314	0.398	0.409	0.282	0.291	0.341	0.384	0.324	0.330	0.355	0.282	0.280	0.292	**0.253**										
Henosepilachna pusillanima	0.367	0.375	0.395	0.384	0.388	0.397	0.382	0.370	0.400	0.399	0.423	0.358	0.411	0.412	0.295	0.383	0.378	0.380	0.395	0.430									
Henosepilachna vigintioctopunctata	0.340	0.344	0.377	0.352	0.360	0.372	0.372	0.350	0.363	0.360	0.405	0.329	0.393	0.403	0.279	0.354	0.350	0.356	0.389	0.399	**0.199**								
Hippodamia convergens	0.239	0.248	0.276	0.260	0.252	0.288	0.381	0.359	0.251	0.256	0.317	0.354	0.285	0.305	0.331	0.259	0.254	0.266	0.269	0.277	0.393	0.383							
Hippodamia variegata	0.256	0.248	0.285	0.266	0.260	0.287	0.399	0.375	0.257	0.263	0.327	0.359	0.307	0.306	0.335	0.263	0.259	0.272	0.279	0.296	0.407	0.386	**0.220**						
Lemnia saucia	0.244	0.217	0.278	0.215	0.213	0.281	0.363	0.348	0.258	0.259	0.311	0.341	0.301	0.294	0.319	0.258	0.250	0.242	0.284	0.294	0.380	0.354	0.274	0.265					
Propylea japonica	0.229	0.212	0.256	0.208	0.207	0.257	0.354	0.336	0.244	0.247	0.309	0.328	0.279	0.286	0.304	0.237	0.234	0.230	0.276	0.275	0.368	0.349	0.260	0.261	0.169				
Propylea sp. *	0.231	0.211	0.260	0.214	0.206	0.263	0.356	0.343	0.242	0.253	0.305	0.337	0.282	0.292	0.312	0.245	0.237	0.229	0.278	0.284	0.378	0.352	0.270	0.260	0.166	**0.111**			
Subcoccinella vigintiquatuorpunctata **	0.344	0.345	0.370	0.350	0.352	0.375	0.356	0.333	0.360	0.365	0.411	0.328	0.395	0.396	0.227	0.354	0.353	0.359	0.380	0.392	0.319	0.290	0.366	0.378	0.363	0.344	0.345		
Dastarcus helophoroides	0.370	0.378	0.391	0.391	0.400	0.405	0.356	0.386	0.403	0.411	0.444	0.377	0.435	0.444	0.356	0.391	0.379	0.408	0.425	0.436	0.411	0.398	0.409	0.421	0.385	0.378	0.382	0.396	
Gloesoma sp.	0.366	0.376	0.407	0.391	0.397	0.404	0.377	0.393	0.398	0.405	0.444	0.366	0.422	0.433	0.334	0.371	0.370	0.401	0.404	0.436	0.395	0.386	0.410	0.417	0.385	0.375	0.398	0.369	0.367

The color indicates rage score ■ >0–0.099 ■0.10–0.199 ■0.20–0.299 ■0.30–0.399 ■0.40–0.449. Numbers in bold indicated pairwise sequence comparisons between species of the same genera. * One rRNA was not sequenced and ** both rRNA were not sequenced. See the respective GENBANK codes and references in Table 1.
