# Supplementary material for: New Light on Historical Specimens Reveals a New Species of Ladybird (Coleoptera: Coccinellidae): Morphological, Museomic, and Phylogenetic Analyses
Source: Insects. 2020 Nov 6;11(11):766. doi: 10.3390/insects11110766 (PMC7694756; doi:10.3390/insects11110766)
Supplement: Supplementary file 1 [file insects-11-00766-s001.zip › Supplementary_files_FINAL-VERSION_970082/Figure S4 _FINAL-VERSION_970082.pdf]

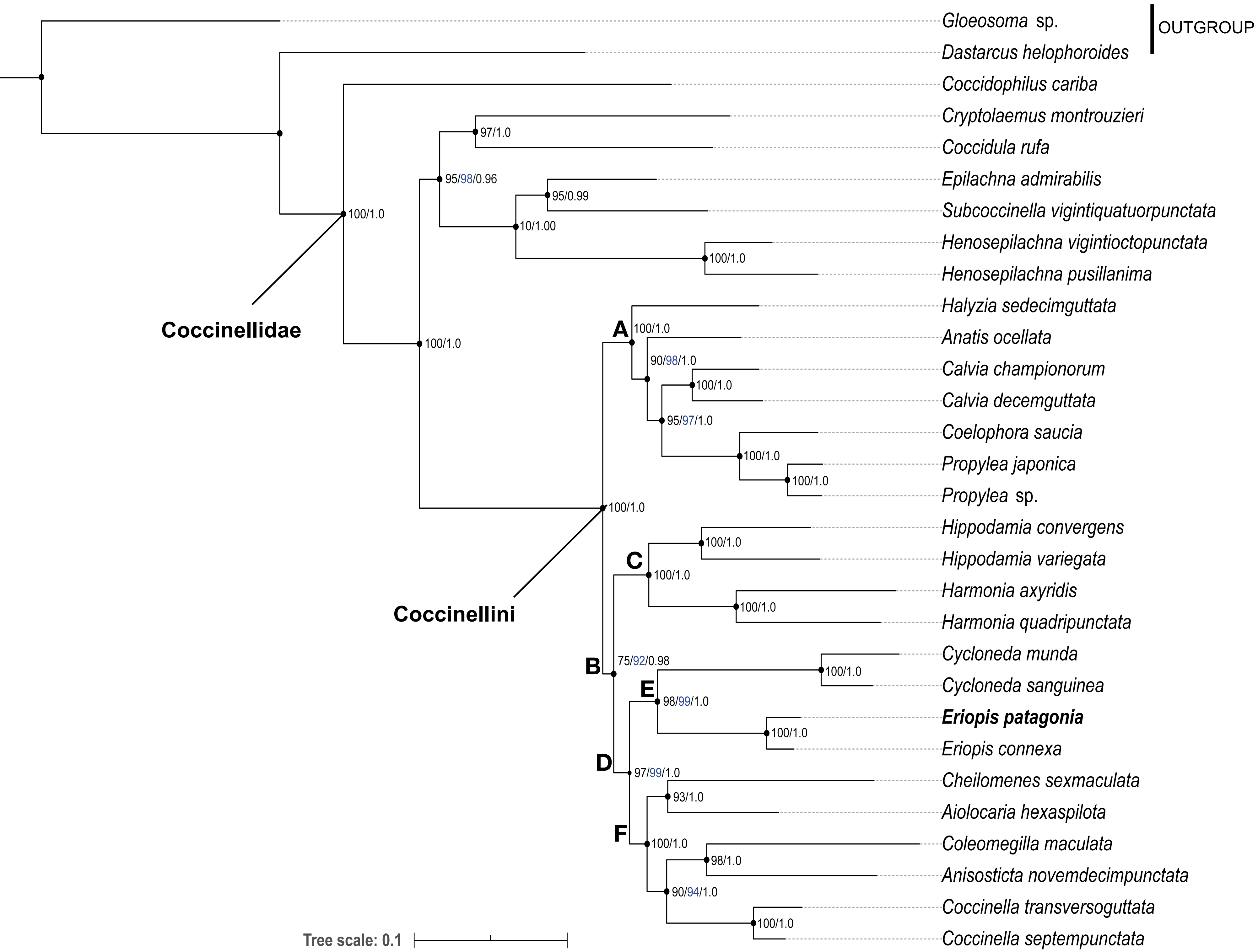

Figure S4. Phylogenetic relationships of Coccinellidae. Tree inferred by the maximum likelihood estimation method (ML) based on 13 protein-coding genes excluding the third-codon positions and two ribosomal RNAs (PCG12_RNA) from mitochondrial genomes of 28 Coccinellidae and two out-groups. ML bootstrap support values and posterior probability values are indicated at the right of each node. Transfer bootstrap expectation value (blue) is only shown when it differs from the bootstrap value.
