# Supplementary material for: New Light on Historical Specimens Reveals a New Species of Ladybird (Coleoptera: Coccinellidae): Morphological, Museomic, and Phylogenetic Analyses
Source: Insects. 2020 Nov 6;11(11):766. doi: 10.3390/insects11110766 (PMC7694756; doi:10.3390/insects11110766)
Supplement: Supplementary file 1 [file insects-11-00766-s001.zip › Supplementary_files_FINAL-VERSION_970082/Table S1__FINAL-VERSION_970082.pdf]

Table S1A. The partition schemes and best substitution models proposed by PartitionFinder for data set PCG_RNA: 13 protein-coding genes including all codon positions combined with the two ribosomal RNA genes

Subset	Partition names	N° of sites	Model
1	12S, NAD4_pos1, NAD4L_pos1, NAD1_pos1, NAD5_pos1	2,173	TVM+G+I
2	16S	1,254	GTR+G+I
3	COX3_pos1, COX2_pos1, ATP6_pos1, CYTB_pos1	1,097	GTR+G+I
4	COX1_pos2, COX2_pos2, CYTB_pos2, COX3_pos2, ATP6_pos2	1,609	TVM+G+I
5	CYTB_pos3, ATP6_pos3, NAD3_pos3, NAD6_pos3, ATP8_pos3	954	TRN+G+I
6	ATP8_pos2, ATP8_pos1, NAD2_pos1, NAD3_pos1, NAD6_pos1	744	GTR+G+I
7	COX1_pos1	512	GTR+G+I
8	COX1_pos3, COX3_pos3	773	HKY+G
9	NAD2_pos3, COX2_pos3	565	HKY+G
10	NAD1_pos2, NAD4L_pos2, NAD4_pos2, NAD5_pos2	1,432	GTR+G+I
11	NAD4L_pos3, NAD1_pos3, NAD4_pos3, NAD5_pos3	1,432	HKY+G+I
12	NAD3_pos2, NAD2_pos2, NAD6_pos2	622	TVM+G+I

Abbreviations used in the Best Model: GTR: General-Time-Reversible model; *HKY*: Hasegawa, Kishino and Yano model; *TRN*: TamuraNei model; *TVM*: Tamura–Nei and the transversion model.
I: invariant sites; *G*: discrete Gamma distribution.

Table S1B. The partition schemes and best substitution models proposed by PartitionFinder for data set PCG12_RNA: 13 protein-coding genes excluding the third-codon positions combined with the two ribosomal RNA genes

Subset	Partition names	N° of sites	Model
1	12S, NAD4L_pos1, NAD4_pos1, NAD5_pos1, NAD1_pos1	2,172	TVM+G+I
2	16S	1,254	GTR+G+I
3	COX2_pos1, COX3_pos1, CYTB_pos1, ATP6_pos1	1,097	GTR+G+I
4	COX1_pos2, COX2_pos2, CYTB_pos2, ATP6_pos2, COX3_pos2	1,607	TVM+G+I
5	NAD2_pos1, NAD6_pos1, NAD3_pos1, ATP8_pos1, ATP8_pos2	740	GTR+G+I
6	COX1_pos1	512	GTR+G+I
7	NAD1_pos2, NAD4L_pos2, NAD5_pos2, NAD4_pos2	1,428	GTR+G+I
8	NAD3_pos2, NAD6_pos2, NAD2_pos2	619	TVM+G+I

Abbreviations used in the Best Model: GTR: General-Time-Reversible model; *TVM*: Tamura–Nei and the transversion model.
I: invariant sites; *G*: discrete Gamma distribution.

Table S1C. The partition schemes and best substitution models proposed by PartitionFinder for data set PCG_AA: 13 protein-coding genes translate into amino acids

Subset	Partition names	N° of sites	Model
1	COX1, COX3, ATP6, CYTB, COX2	1,608	MTART+G+F
2	ATP8, NAD6, NAD3, NAD2	682	MTART+G+F
3	NAD1, NAD4, NAD4L, NAD5	1,430	MTART+G+F

Abbreviations used in the Best Model: MTART: Mitochondrial Arthropoda.
F: empirical base frequencies; *G*: discrete Gamma distribution.
