# Supplementary material for: New Light on Historical Specimens Reveals a New Species of Ladybird (Coleoptera: Coccinellidae): Morphological, Museomic, and Phylogenetic Analyses
Source: Insects. 2020 Nov 6;11(11):766. doi: 10.3390/insects11110766 (PMC7694756; doi:10.3390/insects11110766)
Supplement: Supplementary file 1 [file insects-11-00766-s001.zip › Supplementary_files_FINAL-VERSION_970082/Table S3__FINAL-VERSION_970082pdf.pdf]

Table S3. Estimates of evolutionary divergence between pairwise mitochondrial sequences of Coccinelloidea species.

The value is the number of base substitutions per site between nucleotide sequences estimated by the Kimura-2-parameter model, and the variation rate among sites was modeled with a gamma distribution. The analysis was based on 9452 positions that comprise the PCGs with the three-codon positions for 30 taxa. The out-groups (in blue) are included for comparision with the in-group (Coccinellidae).

**Species names**	*Aiolocaria hexaspilota*	*Anatis ocellata*	*Anisosticta novemdecimpunctata*	*Calvia championorum*	*Calvia decemguttata*	*Cheilomenes sexmaculata*	*Coccidophilus cariba*	*Coccidula rufa* **	*Coccinella septempunctata*	*Coccinella transversoguttata*	*Coleomegilla maculata*	*Cryptolaemus montrouzieri*	*Cycloneda munda* *	*Cycloneda sanguinea*	*Epilachna admirabilis*	*Eriopis connexa*	***Eriopis patagonia***	*Halyzia sedecimguttata*	*Harmonia axyridis*	*Harmonia quadripunctata*	*Henosepilachna pusillanima*	*Henosepilachna vigintioctopunctata*	*Hippodamia convergens*	*Hippodamia variegata*	*Lemnia saucia*	*Propylea japonica*	*Propylea* sp.	*Subcoccinella vigintiquatuorpunctata* **	*Dastarcus helophoroides*
Anatis ocellata	0.215																												
Anisosticta novemdecimpunctata	0.249	0.275																											
Calvia championorum	0.227	0.192	0.270																										
Calvia decemguttata	0.231	0.192	0.277	**0.169**																									
Cheilomenes sexmaculata	0.235	0.260	0.281	0.266	0.263																								
Coccidophilus cariba	0.366	0.367	0.383	0.373	0.373	0.392																							
Coccidula rufa	0.345	0.346	0.376	0.344	0.349	0.382	0.363																						
Coccinella septempunctata	0.216	0.233	0.257	0.236	0.236	0.263	0.372	0.372																					
Coccinella transversoguttata	0.220	0.236	0.269	0.243	0.241	0.261	0.385	0.376	**0.126**																				
Coleomegilla maculata	0.296	0.313	0.311	0.307	0.321	0.318	0.420	0.417	0.266	0.283																			
Cryptolaemus montrouzieri	0.328	0.336	0.367	0.348	0.346	0.361	0.359	0.335	0.352	0.357	0.390																		
Cycloneda munda	0.260	0.274	0.309	0.286	0.282	0.299	0.395	0.401	0.284	0.283	0.336	0.391																	
Cycloneda sanguinea	0.264	0.280	0.311	0.290	0.287	0.309	0.401	0.407	0.281	0.291	0.348	0.393	**0.160**																
Epilachna admirabilis	0.295	0.311	0.331	0.315	0.320	0.342	0.315	0.296	0.325	0.336	0.368	0.284	0.364	0.366															
Eriopis connexa	0.217	0.224	0.259	0.234	0.240	0.255	0.368	0.359	0.228	0.236	0.304	0.343	0.267	0.277	0.310														
Eriopis patagonia	0.212	0.227	0.261	0.237	0.237	0.260	0.370	0.358	0.229	0.233	0.301	0.336	0.265	0.275	0.310	**0.084**													
Halyzia sedecimguttata	0.234	0.204	0.281	0.223	0.220	0.273	0.384	0.353	0.248	0.251	0.330	0.355	0.290	0.299	0.318	0.248	0.249												
Harmonia axyridis	0.269	0.276	0.310	0.282	0.288	0.309	0.399	0.399	0.284	0.280	0.345	0.379	0.321	0.323	0.358	0.291	0.283	0.290											
Harmonia quadripunctata	0.274	0.274	0.311	0.288	0.285	0.317	0.405	0.410	0.286	0.294	0.347	0.386	0.326	0.332	0.360	0.283	0.280	0.295	**0.257**										
Henosepilachna pusillanima	0.373	0.380	0.403	0.388	0.392	0.404	0.385	0.375	0.408	0.407	0.430	0.360	0.413	0.421	0.296	0.389	0.386	0.386	0.402	0.433									
Henosepilachna vigintioctopunctata	0.344	0.348	0.385	0.353	0.362	0.378	0.371	0.352	0.369	0.365	0.409	0.329	0.395	0.408	0.279	0.359	0.355	0.361	0.393	0.402	0.197								
Hippodamia convergens	0.241	0.245	0.278	0.258	0.252	0.292	0.388	0.364	0.251	0.255	0.323	0.357	0.286	0.305	0.335	0.261	0.256	0.266	0.275	0.279	0.399	0.386							
Hippodamia variegata	0.261	0.251	0.291	0.268	0.262	0.294	0.408	0.378	0.263	0.268	0.329	0.364	0.309	0.309	0.342	0.269	0.264	0.275	0.282	0.300	0.414	0.392	**0.224**						
Lemnia saucia	0.248	0.220	0.284	0.219	0.217	0.288	0.373	0.352	0.263	0.264	0.313	0.345	0.302	0.297	0.322	0.264	0.255	0.246	0.287	0.298	0.387	0.359	0.277	0.266					
Propylea japonica	0.230	0.210	0.258	0.208	0.208	0.261	0.363	0.339	0.246	0.247	0.313	0.329	0.279	0.285	0.308	0.240	0.236	0.230	0.281	0.275	0.372	0.351	0.258	0.264	0.172				
Propylea sp.	0.236	0.213	0.265	0.217	0.211	0.270	0.366	0.348	0.248	0.257	0.306	0.340	0.282	0.295	0.317	0.250	0.241	0.233	0.280	0.287	0.385	0.357	0.272	0.261	0.165	**0.114**			
Subcoccinella vigintiquatuorpunctata	0.348	0.349	0.376	0.350	0.355	0.380	0.359	0.336	0.363	0.370	0.417	0.327	0.398	0.401	0.228	0.357	0.356	0.361	0.385	0.398	0.318	0.288	0.369	0.384	0.369	0.347	0.352		
Dastarcus helophoroides	0.376	0.384	0.400	0.396	0.407	0.411	0.361	0.391	0.410	0.416	0.449	0.380	0.437	0.451	0.360	0.400	0.387	0.413	0.433	0.441	0.413	0.401	0.417	0.429	0.395	0.386	0.392	0.400	
Gloesoma sp.	0.371	0.379	0.412	0.393	0.398	0.409	0.382	0.399	0.403	0.409	0.449	0.370	0.425	0.441	0.338	0.376	0.374	0.403	0.408	0.440	0.400	0.391	0.414	0.421	0.390	0.371	0.380	0.401	0.371

The color indicates rage score ■ >0–0.09 ■0.10–0.199 ■0.20–0.299 ■0.30–0.399 ■0.40–0.449. Numbers in bold indicated pairwise sequence comparisons between species of the same genera. See the respective GENBANK codes and references in Table 1.
