# Supplementary material for: New Light on Historical Specimens Reveals a New Species of Ladybird (Coleoptera: Coccinellidae): Morphological, Museomic, and Phylogenetic Analyses
Source: Insects. 2020 Nov 6;11(11):766. doi: 10.3390/insects11110766 (PMC7694756; doi:10.3390/insects11110766)
Supplement: Supplementary file 1 [file insects-11-00766-s001.zip › Supplementary_files_FINAL-VERSION_970082/Figure S3 _FINAL-VERSION_970082.pdf]

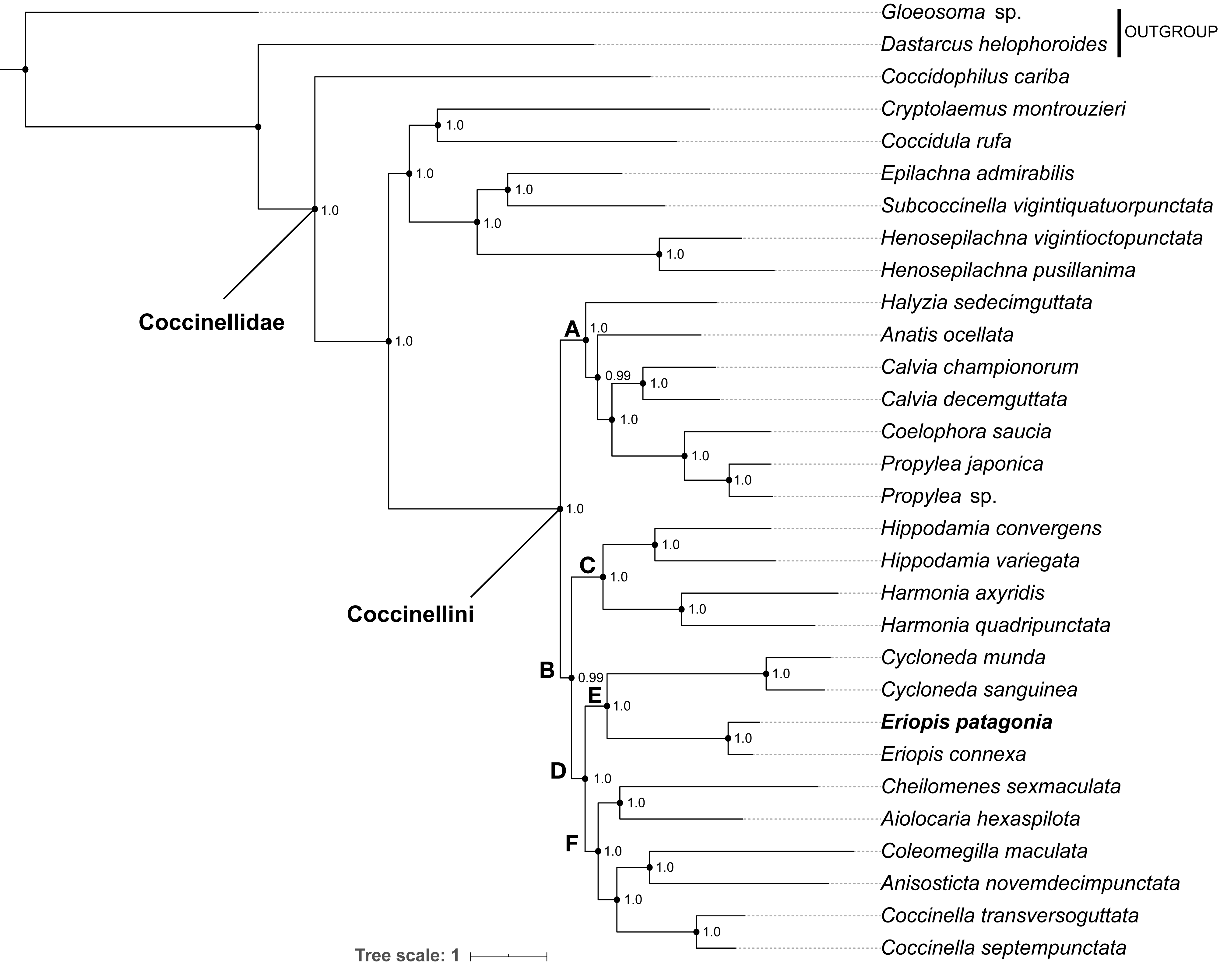

Figure S3. Phylogenetic relationships of Coccinellidae. Consensus tree inferred by the Bayesian Inference (BI) method based on 13 protein-coding genes including all codon positions and two ribosomal RNAs (PCG_RNA) from mitochondrial genomes of 28 Coccinellidae and two out-groups. Bayesian posterior probability values are indicated at the right of each node.
