# Supplementary figures and images for: New Light on Historical Specimens Reveals a New Species of Ladybird (Coleoptera: Coccinellidae): Morphological, Museomic, and Phylogenetic Analyses
Source: Insects. 2020 Nov 6;11(11):766. doi: 10.3390/insects11110766 (PMC7694756; doi:10.3390/insects11110766)

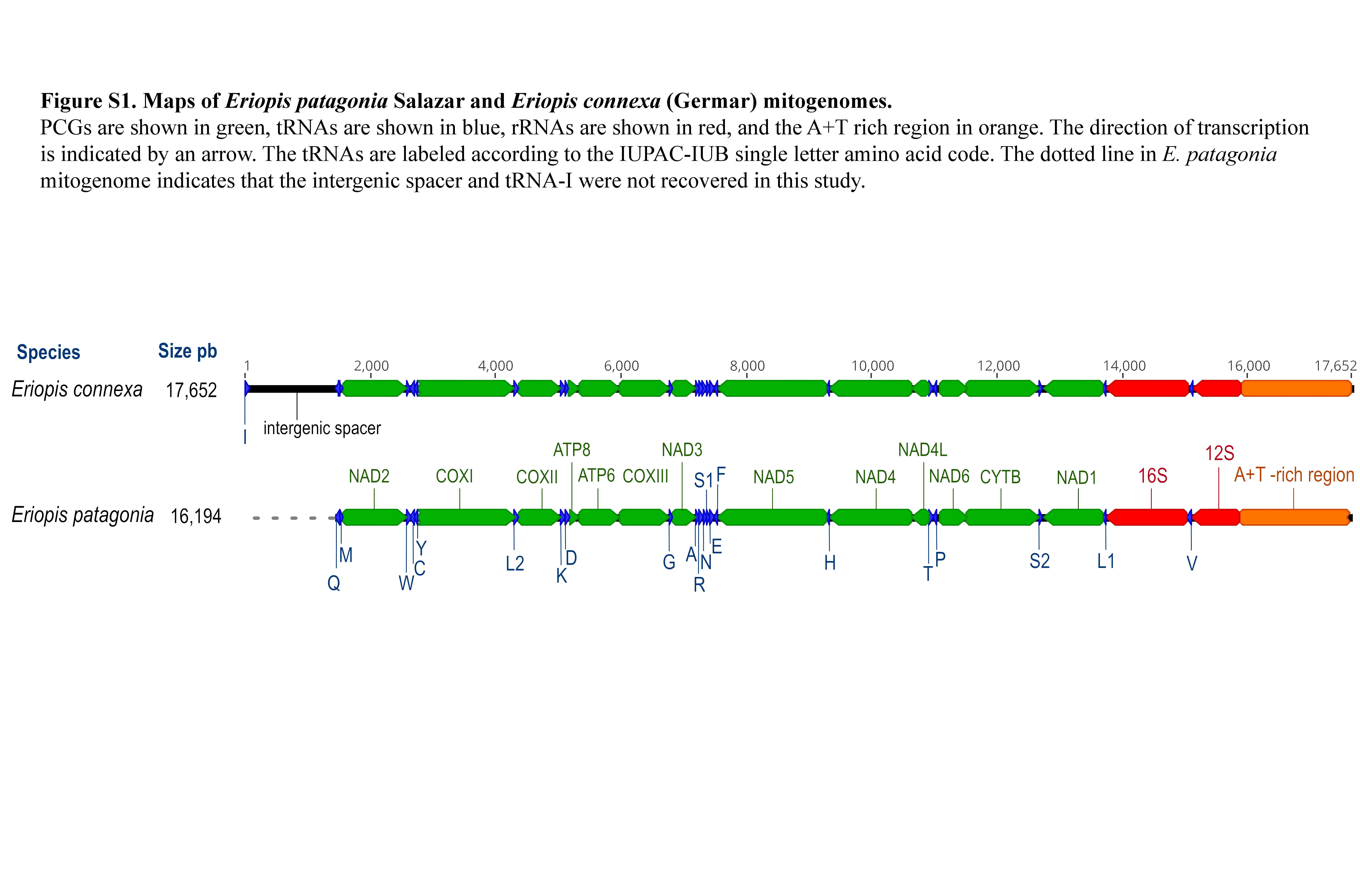

Supplement: Supplementary file 1 [file insects-11-00766-s001.zip › Supplementary_files_FINAL-VERSION_970082/Figure S1_FINAL-VERSION_970082.tiff]

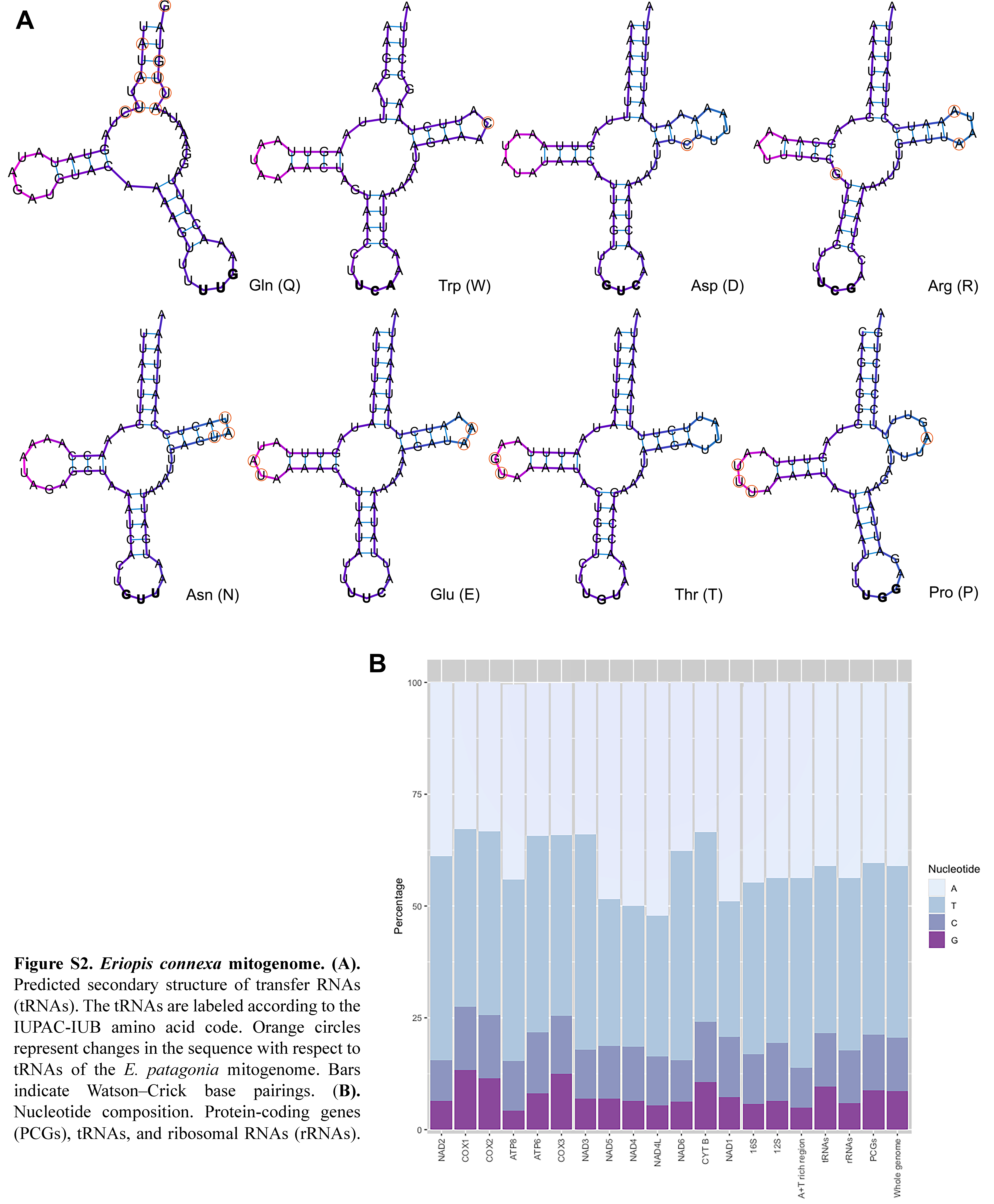

Supplement: Supplementary file 1 [file insects-11-00766-s001.zip › Supplementary_files_FINAL-VERSION_970082/Figure S2__FINAL-VERSION_970082.tiff]
